# A virus carries a gene encoding juvenile hormone acid methyltransferase, a key regulatory enzyme in insect metamorphosis

**DOI:** 10.1038/s41598-017-14059-8

**Published:** 2017-10-19

**Authors:** Jun Takatsuka, Madoka Nakai, Tetsuro Shinoda

**Affiliations:** 1 Forestry and Forest Products Research Institute, Forest Research and Management Organization, Tsukuba, Ibaraki, Japan; 2 Institute of Agriculture, Tokyo University of Agriculture and Technology, Fuchu, Tokyo, Japan; 3 Institute of Agrobiological Sciences, National Agriculture and Food Research Organization, Tsukuba, Ibaraki, Japan

## Abstract

Microbial parasitism, infection, and symbiosis in animals often modulate host endocrine systems, resulting in alterations of phenotypic traits of the host that can have profound effects on the ecology and evolution of both the microorganisms and their hosts. Information about the mechanisms and genetic bases of such modulations by animal parasites is available from studies of steroid hormones. However, reports involving other hormones are scarce. We found that an insect virus, a betaentomopoxvirus, encodes a juvenile hormone acid methyltransferase that can synthesize an important insect hormone, the sesquiterpenoid juvenile hormone. Phylogenetic analysis suggested that this gene is of bacterial origin. Our study challenges the conventional view that functional enzymes in the late phase of the juvenile hormone biosynthesis pathway are almost exclusive to insects or arthropods, and shed light on juvenoid hormone synthesis beyond Eukaryota. This striking example demonstrates that even animal parasites having no metabolic pathways for molecules resembling host hormones can nevertheless influence the synthesis of such hormones, and provides a new context for studying animal parasite strategies in diverse systems such as host-parasite, host-symbiont or host-vector-parasite.

## Introduction

Parasites, pathogens, and symbionts (simplified hereafter to parasites unless otherwise mentioned) use other organisms to perpetuate themselves in nature. Therefore, parasites are likely to have evolved particular strategies to utilize their hosts for completing their life cycles and enhancing their transmission. Recent work on both plant-parasite and animal-parasite interactions suggests that host endocrine systems are important targets that parasites manipulate for their reproductive success^1,2^. In plants, where hormones are involved in signalling for the activation of defence genes and for growth, many parasites adopt the strategies of interference in signalling pathways and of production of plant hormones or their analogues to modulate plant defences and plant growth or structures, thereby increasing infection and transmission success^1^. Hormones are also known to affect immune systems in animals^2^, and there is growing evidence that hormone signalling has a crucial regulatory role for immune system activity in animals^3,4^. Although hormonal modulation as a strategy of animal parasites to evade host immunity is not well studied, it has often been reported that infection by animal parasites perturbed the host hormonal milieu, which may benefit parasites^2,5^. In addition, recent research has found that animal parasites can utilize host hormones for their own survival or reproduction^2^. Elucidation of the genetic bases and mechanisms of host hormonal manipulations is important for unravelling the strategies deployed by host and parasite in their interactions. Limited information comes from research on steroid hormones, primarily sex steroid hormones, where parasites are capable of manipulating steroid hormone signalling by mechanisms such as hormone production, catabolism, or hormone receptor expression^2^. However, comparable information beyond animal parasite-steroid hormone systems, particularly in systems where the parasite and host are phylogenetically unrelated, is scarce.

Hormones are biochemical molecules that regulate specific physiological functions critical to the life cycle such as development, reproduction, and behaviour. During development in holometabolous insects, regulation of the levels of a steroid hormone, 20-hydroxyecdysone (20E), and a sesquiterpenoid hormone, juvenile hormone (JH), governs normal larval development and metamorphosis. The increased level of 20E in the presence of JH induces a larval-larval moult, whereas 20E in the absence of JH provides the signal for metamorphosis (larval-pupal moult). 20E thus induces moulting and JH functions to maintain the larval state^6,7^.

**Figure 1:**
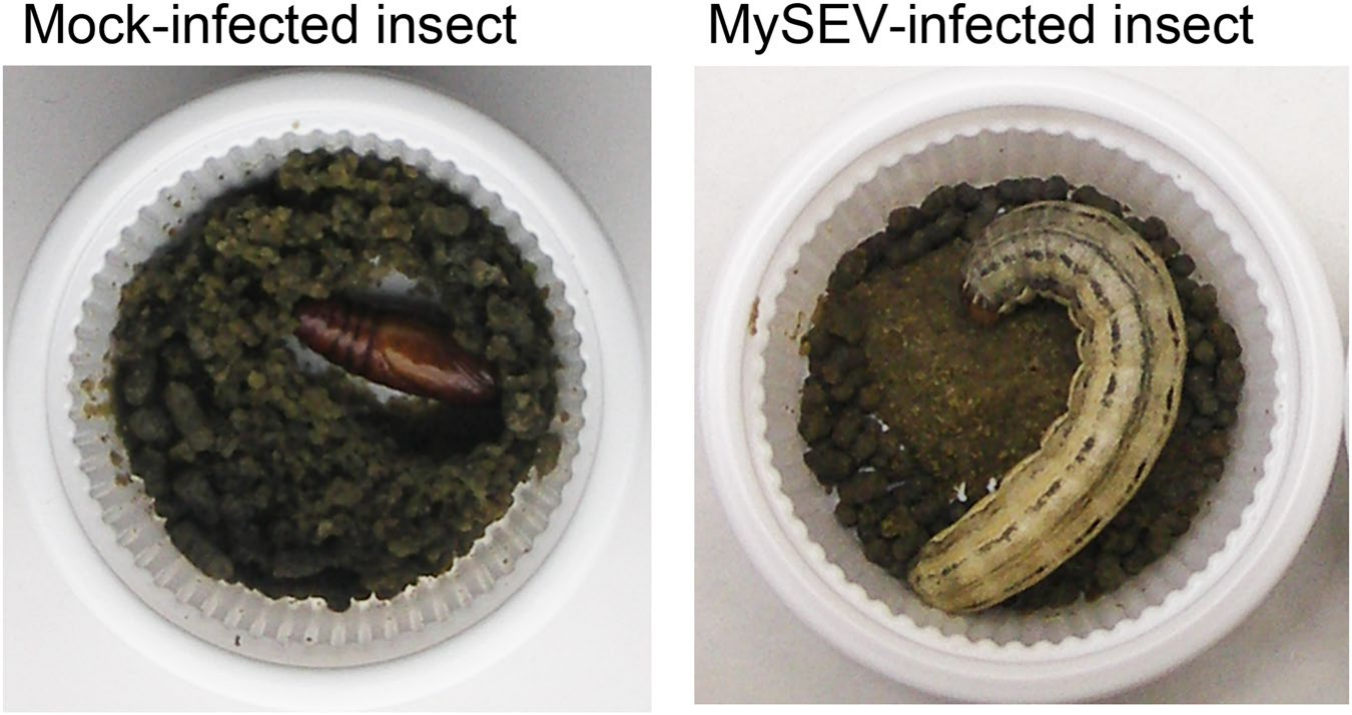
Developmental status of mock- and MySEV-infected *Mythimna separata*. Insects were mock- or MySEV-inoculated as 4th instar larvae and are shown 15 days later in the left and right panels, respectively. The mock-infected larva has pupated.

Entomopoxviruses (EPVs) are insect viruses with a double-stranded DNA genome belonging to the family *Poxviridae* ^8^. Striking characteristics of the pathological effects of EPV infection on the host are a prolonged larval period in the final instar and the inhibition of metamorphosis to pupae^9–11^ (e.g., Fig. 1). These phenotypic characteristics of EPV infections may be advantageous for EPV transmission in host insect populations via increased virus production or inhibition of pupation-associated behaviour^10^. Similar pathological phenotypes have been reported for insect viruses in the family *Baculoviridae* ^12^, and for an insect-pathogenic fungus in the family Clavicipitaceae, *Metarhizium rileyi* ^13^. These pathogens convert 20E to inactive forms by encoding the enzymes ecdysteroid UDP-glucosyltransferase and ecdysteroid 22-oxidase, respectively, resulting in inhibition of moulting. However, no genetic evidence for such enzymes targeting 20E has been found in EPV genomes. Previous studies indicated higher JH titres in the haemolymph of larvae infected with EPVs belonging to the genus *Betaentomopoxvirus* (EPVs infecting lepidopteran insects) than in non-infected larval haemolymph^10,11^. A survey using available genome sequence data of five *Betaentomopoxvirus* species revealed that they possessed a plausible candidate gene responsible for this JH titre elevation. We found that all five EPV genomes encoded homologues of S-adenosyl-L-methionine (SAM)-dependent methyltransferase (MTase). SAM-dependent MTase belongs to an enzyme family having diverse physiological functions^14^, including JH biosynthesis in insects. The JH biosynthesis pathway is divided into early and late phases, and the late phase is thought to be unique to insects or arthropods except for a very few poorly studied examples in plants^15,16^. In the last steps of the JH biosynthetic pathway in insects, farnesoic acid (FA) is converted to active JH by epoxidation and methylation. In lepidopteran insects, it is thought that FA is first converted to JH acid (JHA) by a P450 monooxygenase, and JHA then becomes active JH by methylation by an SAM-dependent MTase called JHA methyltransferase (JHAMT). In insects of the orders Blattodea and Orthoptera, FA is thought to be methylated first, followed by epoxidation, resulting in active JH. Regulation of *JHAMT* is thought to play a crucial role in the biosynthetic activity of JH in the corpora allata, the primary organ for *de novo* JH biosynthesis^17^.

Here, we report that SAM-dependent MTase encoded in the genome of a betaentomopoxvirus, Mythimna separata entomopoxvirus (MySEV), is a functional JHAMT, and that the viral *JHAMT* is likely to be descended from a member of the domain Bacteria.

## Results

### MySEV SAM-dependent MTase has homologies with insect JHAMTs

**Figure 2:**
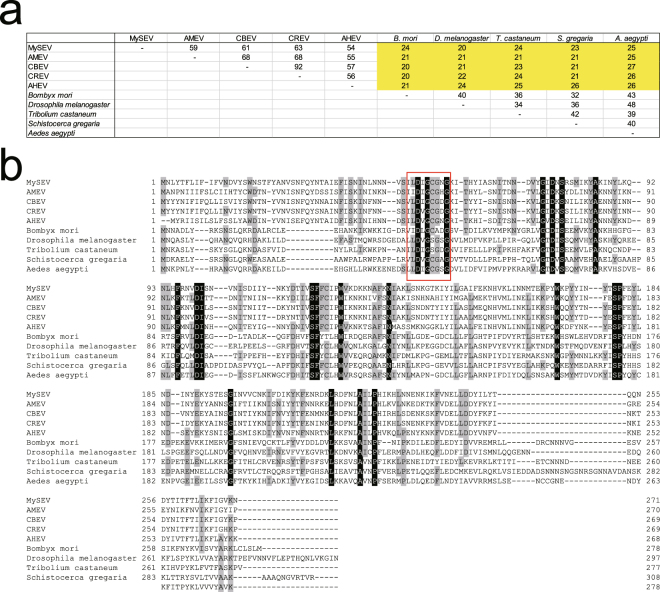
Similarities among SAM-dependent MTases of betaentomopoxviruses and selected insect JHAMTs. (**a**) Sequence identity (%) matrix for the betaentomopoxvirus SAM-dependent MTases and the insect JHAMTs. (**b**) Alignment of the betaentomopoxvirus SAM-dependent MTases and the insect JHAMTs. Amino acids common to all sequences are indicated by white letters on a black background, and those common to more than half of the sequences are indicated by grey-shadowed letters. The red box indicates the putative SAM-binding motif. The following sequences were used: MySEV, Mythimna separata entomopoxvirus (CCU56237); AMEV, Amsacta moorei entomopoxvirus (NP_064786); CBEV, Choristoneura biennis entomopoxvirus (YP_008004135); CREV, Choristoneura rosaceana entomopoxvirus (YP_008004449); AHEV, Adoxophyes honmai entomopoxvirus (YP_008003847); *Aedes aegypti* (XP_001651876); *Bombyx mori* (NP_001036901); *Drosophila melanogaster* (NP_609793); *Tribolium castaneum* (NP_001120783); and *Schistocerca gregaria* (ADV17350).

SAM-dependent MTase of MySEV had marked similarities with MTases of other betaentomopoxviruses, invertebrate iridescent virus 6, Bacteria, and Archaea, with high alignment scores. Significant similarities with insect SAM-dependent MTases were also observed, although the alignment scores were lower than those with MTases from viruses, Bacteria, and Archaea (see Supplementary Table S1). Amino acid identities of 20–27% with selected insect JHAMTs, which have been functionally characterized, were observed (Fig. 2a). SAM-dependent MTases of EPVs and the insect JHAMTs have several conserved amino acid regions including the putative SAM-binding motif (Fig. 2b).

### MySEV SAM-dependent MTase gene encodes a JHAMT

**Figure 3:**
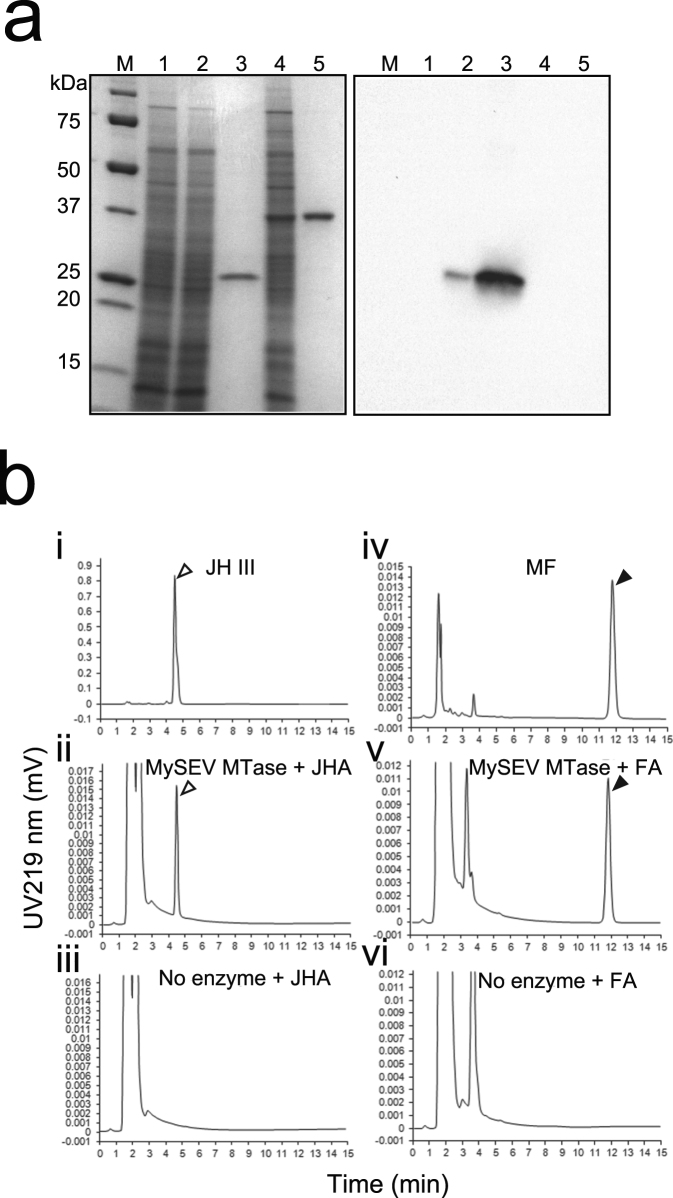
MySEV SAM-dependent MTase gene encodes a JHAMT. (**a**) SDS-PAGE and Western blotting analyses of His-tagged recombinant MySEV SAM-dependent MTase and *Mythimna separata* JHAMT expressed using the baculovirus protein expression system. SDS–PAGE and Western blotting analyses are shown in the left and right panels, respectively. Each image in the panels was cropped from different exposures. Exposure for gel image was optimized for intense bands to prevent saturation (overexposure) (see Supplemental Fig. S1 for a full-length gel image, and full-length blot images acquired with different exposure times). An anti-MySEV SAM-dependent MTase peptide antiserum was used for Western blotting analysis. Lane 1, Lysate of cells infected with viruses that have no target gene. Lane 2, Lysate of cells infected with viruses expressing the MySEV SAM-dependent MTase gene. Lane 3, Purified His-tagged MySEV SAM-dependent MTase protein. Lane 4, Lysate of cells infected with viruses expressing the *M. separata* JHAMT gene. Lane 5, Purified His-tagged *M. separata* JHAMT. Lane M, protein standard marker. Note that the expressed protein has a lower mass than MySEV SAM-dependent MTase protein in larval haemolymph (Fig. 4b). This difference may be attributable to glycosylation of the MySEV SAM-dependent MTase *in vivo*. (**b**) Enzyme assay for JHAMT. JHA or FA metabolites generated with recombinant MySEV SAM-dependent MTase were analysed by reverse phase high-performance liquid chromatography. Standard JH III (i) and methyl farnesoate (MF) (iv). JHA III (ii) or FA (v) were incubated with the purified His-tagged MySEV SAM-dependent MTase. No enzyme was added in the reactions of JHA III (iii) or FA (vi). Open and closed arrowheads indicate peaks corresponding to JH III and MF, respectively.

To determine whether the SAM-dependent MTase gene in the MySEV genome encodes a functional homologue of the SAM-dependent MTases involved in JH biosynthesis, a recombinant MySEV SAM-dependent MTase produced using a baculovirus expression system was examined for its enzymatic activity (Fig. 3a and b). When JHA III or FA were incubated with purified recombinant MySEV SAM-dependent MTase in the presence of SAM, sharp peaks at the retention times corresponding to those of the juvenoid methyl ester standards (JH III and methyl farnesoate) were observed by reverse phase high-performance liquid chromatography. These peaks were absent in the reactions lacking recombinant MySEV SAM-dependent MTase. We concluded that the SAM-dependent MTase gene is a functional homologue of *JHAMT*.

### MySEV SAM-dependent MTase gene is expressed in virus-infected insect tissues and the protein accumulates in the haemolymph

**Figure 4:**
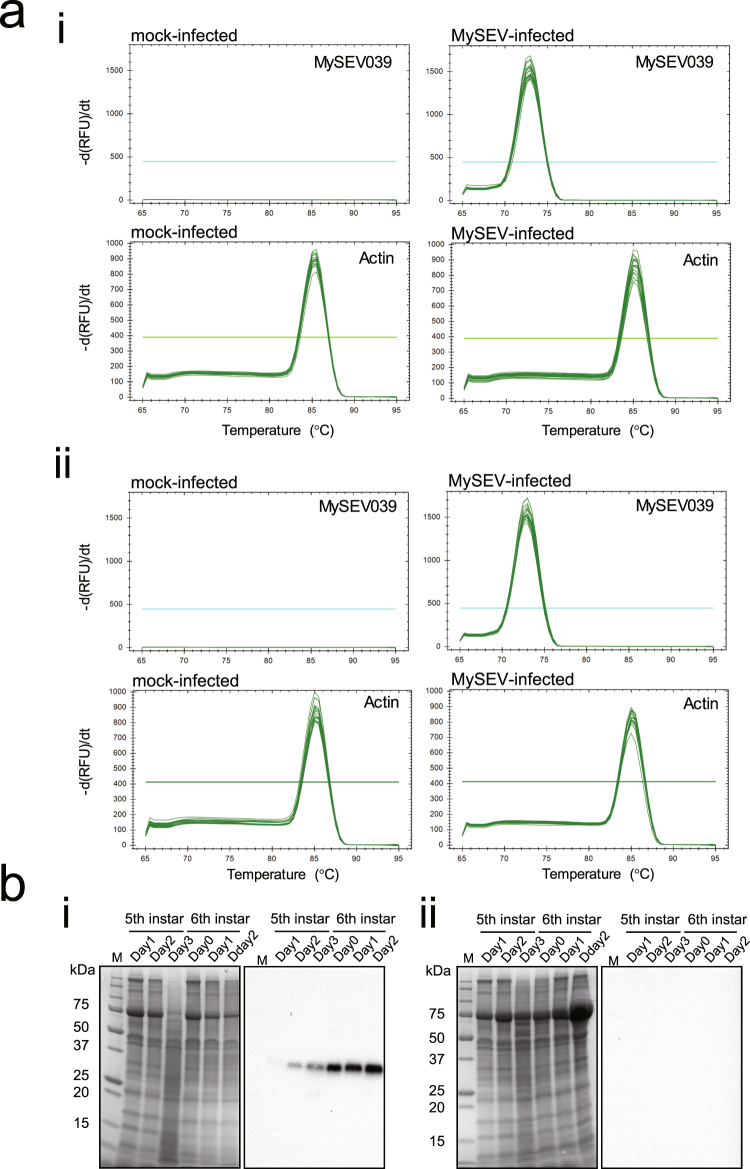
MySEV SAM-dependent MTase gene is expressed in virus-infected insect tissues and the protein accumulates in the haemolymph. (**a**) Analysis of MySEV SAM-dependent MTase gene (ORF name: MySEV039) expression. Larvae were MySEV- or mock-infected at 4th instar. Total RNAs extracted from (i) fat bodies and (ii) haemocytes of either mock-infected or MySEV-infected larvae were subjected to melting curve analysis after RT-PCR. MySEV039, and the *M. separata* β-actin gene as an internal control, were targeted for PCRs as indicated in the panels. Combined profiles for three samples on each of day 1, 2, 3 of 5th instar and day 0, 1, 2 of 6th instar are shown in each panel. Thresholds for significant amplification are shown in each panel (i.e., lines parallel to the X-axes). RFU, relative fluorescence units. No-RT negative control templates yielded no amplification products. (**b**) SDS-PAGE (left panels) and Western blotting (right panels) analyses of MySEV SAM-dependent MTase in the haemolymph of larval *M. separata*. Gel and blot images in the panels were cropped from different parts of the same gel and blot images, respectively. Exposure for gel image was optimized for intense bands to prevent saturation (overexposure) (see Supplemental Fig. S2 for a full-length gel image, and full-length blot images acquired with different exposure times). Larvae were MySEV- or mock-infected at 4th instar, and haemolymph was collected from 5th instar to early 6th instar. Anti-MySEV SAM-dependent MTase peptide antiserum was used for Western blotting analyses. (i) MySEV-infected larvae. (ii) Mock-infected larvae. Lane M, protein standard marker.

We next analysed gene and protein expression under the same conditions used in a previous study where the physiology of MySEV-infected insects, including JH titres, was examined^10^. MySEV was administered to newly moulted 4th instar larvae of the oriental armyworm *Mythimna separata*, and gene and protein expression were analysed from day 1 of 5th instar to early 6th instar (final instar). In accordance with previous results^10^, MySEV-infected insects developed at almost the same rate as mock-infected insects during 4th and 5th instars, but their development was prolonged substantially during the final (6th) instar compared to mock-infected insects; they eventually died as larvae, whereas all control insects pupated (Table 1). Reverse transcription PCR (RT-PCR) followed by melting curve analysis confirmed the expression of the MySEV SAM-dependent MTase gene in MySEV-infected insects throughout the period examined. No expression of the gene was observed in mock-infected insects (Fig. 4a). MySEV SAM-dependent MTase in the haemolymph of MySEV-infected insects was detected by Western blot analysis throughout the period examined. The protein level increased with insect development from day 1 of 5th instar to early 6th instar (Fig. 4b(i)). The anti-MySEV SAM-dependent MTase peptide antiserum used in the analyses did not react to haemolymph of mock-infected insects (Fig. 4b(ii)) or to either cultured cells or *M. separata* JHAMT expressed by a baculovirus expression system (Fig. 3a), indicating that the analyses specifically detected MySEV SAM-dependent MTase.

**Table 1:** Development and pupation of *Mythimna separata* larvae that were mock- or MySEV-infected at 4th instar.

Virus dose administered^1)^	N	Mean development time in days (SEM)^2)^			Total larval period^2),3)^	Frequency of pupation (%)
		Fourth instar	Fifth instar	Sixth instar		
0	62	3.0 (0.0) a	3.1 (0.1) a	7.4 (0.1) a	13.5 (0.1) a	100
10^4^	62	3.0 (0.0) a	3.7 (0.1) b	14.1 (0.2) b	20.7 (0.2) b	0

1) Occlusion bodies/larva.

2) Values followed by different letters are significantly different at *p* < 0.0001 by the Wilcoxson test.

3) Total larval period indicates the interval between administration and pupation or death.

### Host *JHAMT* expression profiles are similar in MySEV- and mock-infected hosts

**Figure 5:**
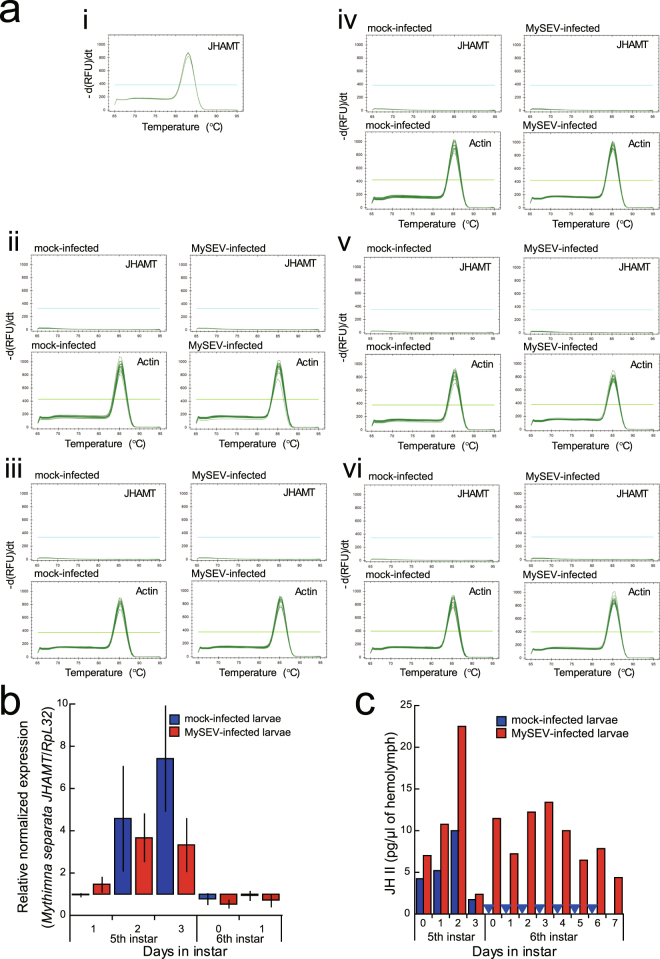
Host *JHAMT* expression profiles are similar in MySEV- and mock-infected hosts. (**a**) *M. separata JHAMT* expression in larval tissues. Larvae were MySEV- or mock-infected at 4th instar. Results of melting curve analyses after RT-PCRs are shown. Examples of positive control for *M. separata JHAMT* expression are shown in (i), where total RNAs extracted from head tissues, in which the corpora allata are located, were analysed. Total RNAs extracted from (ii) fat body, (iii) haemocytes, (iv) gut, (v) Malpighian tubules and (vi) epidermis of either mock-infected or MySEV-infected larvae were analysed for the expression of *M. separata JHAMT*. *M. separata JHAMT*, and the *M. separata* β-actin gene as an internal control, were targeted for PCRs as indicated in the panels. Combined profiles for three samples on each of day 1, 2, 3 of 5th instar and day 0, 1, 2 of 6th instar are shown in each panel. Thresholds for significant amplification are shown in each panel (i.e., lines parallel to the X-axes). RFU, relative fluorescence units. No-RT negative control templates yielded no amplification products. (**b**) Relative normalized expression of the *M. separata JHAMT* in head tissues of larval *M. separata*. Larvae were mock- or MySEV-infected at 4th instar, and heads were collected from 5th instar to early 6th instar. Expression levels were analysed by quantitative RT-PCR and normalized to those of internal *RpL32*. The normalized expression level of day 1 of 5th instar mock-infected larvae is shown as 1. Bars indicate standard errors. The expression levels were affected by days in instar (*p* = 1.0000 × 10^−5^), but not by infection status (i.e., EPV infection vs. mock infection) (*p* = 0.1483), and interaction between infection status and days in instar (*p* = 0.5993). The expression in early 6th instar was downregulated from 5th instar (*p* = 1.2056 × 10^−6^). (**c**) JH titres in haemolymph of larval *M. separata*. Larvae were mock-infected (blue) or infected with MySEV (red) at 4th instar, and JH titres were measured from 5th to 6th instar. Arrowheads indicate that JH titres were below the detection limit. Data are reproduced with permission from Nakai *et al*.^10^.

RT-PCRs of host *JHAMT* from 5th instar to early 6th instar showed no detectable expression in fat body, haemocytes, gut, Malpighian tubules or epidermis in either MySEV- or mock-infected hosts (Fig. 5a). Among the tissues examined, only heads containing the corpora allata showed expression (Fig. 5a(i)). Host *JHAMT* expression levels normalized by those of the ribosomal protein L32 (RpL32) gene were found to be similar between the MySEV- and mock-infected hosts during 5th instar to early 6th instar by quantitative RT-PCR analyses (Fig. 5b). Statistical analysis utilizing generalized linear modelling techniques showed infection status (i.e., EPV infection vs. mock infection), and interaction between infection status and days in instar revealed no significant effect (χ^2^ = 2.0896, *p* = 0.1483 and χ^2^ = 2.7567, *p* = 0.5993 for the former and latter, respectively). Days in instar significantly affected the expression levels (χ^2^ = 28.5011, *p* = 1.0000 × 10^−5^). Notably, despite the marked difference in JH titres between MySEV- and mock-infected larvae in 6th instar (elevated levels vs. no detectable level) (Fig. 5c), *M. separata JHAMT* expression in both MySEV- and mock-infected larvae in early 6th instar was similarly downregulated from 5th instar (Fig. 5b). (Contrast, χ^2^ = 23.5683, *p* = 1.2056 × 10^−6^).

### EPV JHAMTs are clustered with SAM-dependent MTases of an unrelated virus and bacteria

**Figure 6:**
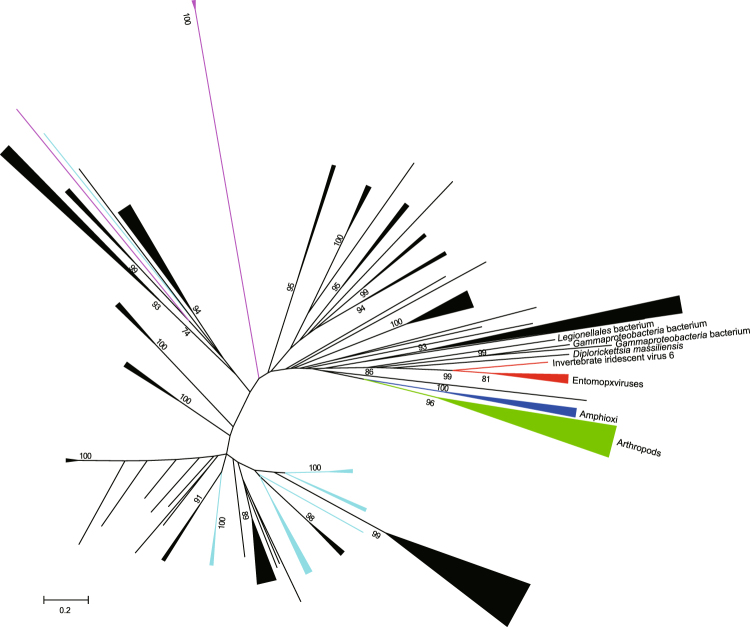
Phylogenetic tree derived from a multiple alignment of homologous proteins with MySEV JHAMT extracted from a BLASTP search result. The tree was inferred from a maximum likelihood analysis. Numbers at the nodes indicate bootstrap values of maximum likelihood analysis generated after 100 replicates. Branches are represented with colors depending on sources: red, viruses; green, arthropods; blue, amphioxi; pink, fungi; black, bacteria; cyan, archaea. Many clusters are compressed (see Supplementary Fig. S4 for a non-compressed tree).

In phylogenetic analysis (Fig. 6), EPV JHAMTs were clustered together with an SAM-dependent MTase of invertebrate iridescent virus 6, an insect virus belonging to the family *Iridoviridae* infectious to many insects^18^, with high bootstrap support values. These viral SAM-dependent MTases were within a clade of SAM-dependent MTases from bacteria: two species of gammaproteobacteria, and two species from the order *Legionellales*, including *Diplorickettsia massiliensis*, an obligate intracellular symbiont of ticks that is also a tick-borne infectious disease agent^19,20^.

## Discussion

Our study demonstrates that MySEV possesses a functional JHAMT gene. This represents the first discovery of a virus gene that enables the synthesis of a host hormone. JH titres in normal insects are regulated by synthesis with JHAMT and catabolism with esterases, mainly with JH esterase (JHE). Several possible mechanisms have been proposed to explain the higher JH titres in EPV-infected insects than in uninfected insects^10,11^. For example, JHE is produced in fat body, which is a major replication tissue for EPV. Thus, EPV infection may suppress JHE production and/or release into the haemolymph. This would result in a reduced JHE titre, leading to incomplete JH catabolism, which in turn would contribute to higher JH titres in infected insects than in uninfected insects. We propose, here, an additional scenario in which betaentomopoxviruses carrying functional *JHAMT*s can convert JHA, including JHA resulting from catabolism by JHE, to active JH. EPV *JHAMT* can thereby contribute to elevated JH titres in infected insects, and this in turn blocks the surge of 20E. Elevated JH titre in the haemolymph reportedly prevents the release of prothoracicotrophic hormone from the brain, which normally induces ecdysteroid release from the prothoracic gland^7^. Therefore, infected insects can be maintained in the larval state.

The expression profile of MySEV JHAMT protein provides a rational explanation for JH titres in the haemolymph of MySEV-infected insects that were administered the virus at 4th instar, if MySEV JHAMT can convert JHA to active JH *in vivo* as we hypothesize. A previous study^10^ showed that JH titres in MySEV-infected insects were higher than those in mock-infected insects, except for the comparable titres on day 3 of 5th instar, just before the moult to final (6th) instar (Fig. 5c), when JH is known to be catabolized in mock-infected larvae. After day 3 of 5th instar, JH titres in MySEV-infected insects increased again and were maintained at elevated levels during 6th instar, while those in mock-infected insects fell below the detection limit. The elevated JH titres in 5th instar to early 6th instar (day 0 of 5th instar to day 1 of 6th instar) cannot be explained by JHE suppression, because JHE titres were similar in mock- and MySEV-infected insects during this period, while JHE in the infected insects was suppressed after day 1 of 6th instar^10^. Our hypotheses for this elevation in JH titres were that MySEV JHAMT synthesized JH from JHA, and/or that MySEV infection stimulated host insects to produce JH in the corpora allata or other tissues. Analyses of host *JHAMT* expression indicated that the elevation in JH titres was not attributable to the latter. Collectively, our data for MySEV JHAMT protein in MySEV-infected larval haemolymph during this period strongly suggest that MySEV JHAMT synthesized JH from JHA, explaining the elevated JH titres in 5th instar to early 6th instar.

Insect JHAMTs are localized in the corpora allata, from which synthesized JH is secreted. There are also reports that JHAMTs are produced in other tissues during specific stages such as the prepupal period, in which JHAMTs are thought to convert JHA to JH within cells^17,21^. The synthesized JH is hypothesized to be used locally in such tissues. Interestingly, MySEV JHAMT is secreted into the haemolymph. EPV replicates mainly in haemocytes and fat body cells, which are not JH-secretory cells in normal insects. Secretion of MySEV JHAMT may be preferable to produce JH from JHA in the haemolymph and to allow the converted JH to act effectively on the whole body of the host.

We believe that MySEV JHAMT is biologically functional for inhibition of host metamorphosis. However, the role may be non-essential, because biological entities often adopt more than one strategy to express a particular phenotype. EPV has at least one other strategy for the elevation of host JH titre, namely suppression of the JH-degrading enzyme JHE. The relative contributions of these strategies should be clarifiable by adopting a reverse genetics system for MySEV.

The phylogenetic analysis reveals that MySEV JHAMT has evolutionary links to the SAM-dependent MTases of invertebrate iridescent virus 6 and bacteria including *D. massiliensis*. Although functional analyses of these SAM-dependent MTases remain to be done, it is worth noting that invertebrate iridescent virus 6 has been reported to have JH-like activity^22,23^. *D. massiliensis* is a recently discovered symbiont of ticks^19^. Closely related *Diplorickettsia spp*. have also been reported from insects^24,25^. Although a juvenile hormone has not been identified in ticks, it may be of value to study the role of this SAM-dependent MTase in the symbiotic interaction. The phylogenetic analysis also reveals that the closest relative of viral SAM-dependent MTase is found in bacteria, suggesting that an ancestral virus acquired the SAM-dependent MTase gene from an ancestral bacterial species.

Our results challenge the conventional view that enzymes in the late phase of the JH biosynthesis pathway are almost exclusive to insects, or to other arthropods where similar biosynthetic pathways are thought to operate, with only a few exceptions reported in plants^15,16^. In crustaceans, SAM-dependent MTase converts FA to the crustacean juvenile hormone, methyl farnesoate^26^. Genes like *JHAMT* that are involved in the synthesis of sesquiterpenoid hormones such as JH and methyl farnesoate may also be distributed and function beyond the Eukaryota (i.e., in Bacteria or Archaea that interact with insects or arthropods), and may thus have a significant impact on the ecology and evolution of the microorganisms and their hosts. JH and methyl farnesoate regulate a wide variety of physiological processes in insects and arthropods, including development, reproduction, sex, behaviour, diapause, and polyphenisms^6,26^. Such microorganisms may be able to increase their own fitness by altering their hosts’ phenotypes through modulation of juvenile hormone titres.

Another facet of the discovery of a virus JHAMT that is more similar to bacterial SAM-dependent MTases than to those of insects is that it provides an opportunity to elucidate the fundamental molecular structure that underlies the function of a JHAMT. Analyses of stereo-structures of insect JHAMT molecules that are specific to a restricted panel of organisms should facilitate the development of growth regulators (pesticides) with high specificity. Structural information about the virus JHAMT will contribute to the design of further specific and biologically safe molecules.

JH production in a eukaryotic microorganism has been reported for a model fungus, *Aspergillus nidulans*, although its genetic basis was not clarified and the pathway appeared to be different from JH biosynthesis in insects^27^. Infections of insects with microsporidian parasites, including *Nosema ceranae*, a putative aetiological agent of colony collapse disorder in honey bees, have been reported to induce elevated JH titres and cause abnormality in host development, metamorphosis, or behaviour^28–32^. There are studies^31,32^ reporting that implantation of *Nosema sp*. into allatectomized insects (i.e., insects whose corpora allata were removed by surgery) led to JH-like effects on insect development, suggesting to the authors that *Nosema sp*. produced JH or JH-like substances. However, the underlying genetic basis of these effects remains unknown. Taken together with the above examples, our study highlights the need for research about JH synthesis in viruses and microorganisms irrespective of their domain of life, and, more widely, tells us that manipulation of the JH signalling pathway by parasites is a research area to be explored.

Some animal parasites that are themselves animals are known to produce the same hormones or analogues as their hosts, as illustrated by the production of JH in insect parasitoids^33^ or of steroid hormones in protozoan or helminth species^34^. However, host hormone production by animal parasites beyond the animal kingdom may be achieved by conversion of metabolites cognate with host hormones by enzymes in their own metabolic systems. Furthermore, the striking example of virus *JHAMT* demonstrated here implies that even animal parasites having no metabolic pathways for molecules cognate with host hormones may nevertheless be capable of producing host hormones by acquiring genes involved in hormone synthesis by horizontal gene transfer via hologenome gene exchange networks^35^, where genes on the genomes of hosts and their interacting microbiome/virome can act as sources for acquiring new phenotypes from each other.

## Methods

### Insects and virus

*Mythimna separata* were originally collected from a maize field in the Tokyo University of Agriculture and Technology Field Science Center, Tokyo, Japan and continuously reared. Larvae were kept at 25 °C with a 16-h photoperiod until pupation, and were reared on an artificial diet^36^. MySEV was originally isolated from *M. separata* larvae^37^. A cloned viral genotype has been approved as a species belonging to the genus *Betaentomopoxvirus*, and its genome has been sequenced in full^38^. The cloned virus was propagated in larval *M. separata* and proteinaceous occlusion bodies (OBs) were purified. EPV produces OBs in which virions are embedded. OBs ingested by host insects dissolve in the midgut and release virions, initiating infection of columnar epithelial cells followed by systemic infection. We thus used MySEV OBs as the infectious unit.

### BLAST search using MySEV SAM-dependent MTase

MySEV SAM-dependent MTase (GenBank accession number CCU56237) was used for a BLASTP search implemented on the BLAST server at the National Center for Biotechnology Information (https://blast.ncbi.nlm.nih.gov/Blast.cgi). Alignment with selected insect JHAMTs, which have been functionally characterized, was carried out using MUSCLE incorporated in GENETYX version 13 (GENETYX).

### Histidine-tagged recombinant protein preparation and enzyme assay

The MySEV SAM-dependent MTase gene sequence (GenBank accession number NC_021246; ORF name, MySEV039), excluding the signal sequence, was PCR-amplified using the primers 5′-cgggatccatgCATCATCATCATCATCATTGGAATTCTACTTTTTACGCTAATG-3′ and 5′-gctctagaTTAATTTTTAACTCCTATAAATTTTATAAGTGTG-3′ (lower-case letters indicate restriction sites; underline represents the His-tag sequence.). The amplicon was cloned into the BamHI-XbaI site of pFastBac1 vector (Thermo Fisher Scientific). Recombinant histidine-tagged protein was expressed using the Bac-to-Bac baculovirus expression system (Thermo Fisher Scientific) and purified using a nickel-nitrilotriacetic acid column. Similarly, the *M. separata* JHAMT gene (GenBank accession number KM926339) was cloned into pFastBac/NT-TOPO (Thermo Fisher Scientific) using the primers 5′-AATAACGCTGTTTTATACGAAAAATGCAAC-3′ and 5′-TCAGACCTGCTTGTTTAATTCTTCCAG-3′, and His-tagged protein was expressed and purified as above. The recombinant MySEV SAM-dependent MTase protein was detected by Western blot analysis. Proteins were subjected to sodium dodecyl sulphate–polyacrylamide gel electrophoresis (SDS–PAGE) analysis and transferred to a polyvinylidene difluoride membrane (Hybond P, GE Healthcare). A polyclonal antiserum against a putative epitope of MySEV SAM-dependent MTase, NDINYEKYSTESGINC, was prepared by immunizing a mouse. Western blot analysis was carried out utilizing the iBind Western System (Thermo Fisher Scientific) with the anti-MySEV SAM-dependent MTase peptide antiserum (1:1000 dilution) and alkaline phosphatase-conjugated anti-mouse IgG (1:1000 dilution), followed by detection of signals by CDP-Star. Gel and blot images were acquired utilizing a molecular imager (ChemiDoc XRS+ Image System, Bio-Rad). The purified recombinant protein was subjected to enzyme assay for JHAMT activity as described previously^17^.

### MySEV SAM-dependent MTase gene expression in insect tissues and protein detection in haemolymph

Insects were bioassayed as described in a previous study in which JH, JHE, and ecdysone titres in larval haemolymph were analysed^10^. Newly moulted fourth-instar larvae were individually allowed to ingest a 1-µl droplet containing 10^4^ OBs of MySEV and 10% sucrose (w/v) in water. As a mock-inoculated control, larvae were fed a 1-µl droplet of the same solution without virus OBs. Larvae that ingested the entire droplet were individually transferred to 1-oz cups containing fresh artificial diet, and reared at 25 °C with a 16-h photoperiod. Sixty-two larvae each from MySEV- and mock-inoculated treatments were set aside to monitor development and mortality at 24-h intervals. Differences in developmental times between MySEV-infected and mock-infected larvae were analysed by the Wilcoxson test. From 1 day after moulting to fifth instar, larval haemocytes, fat bodies, and haemolymph were collected at 24-h intervals.

Fat bodies and haemocytes were processed for RNA extraction using the ReliaPrep RNA Tissue Miniprep System (Promega). Three samples per tissue were analysed for each day of the experiment. For each sample, approximately 10 mg of fat body tissue in total was dissected from two larvae during 5th instar, and from one larva during 6th instar. A total of 100 µl of haemolymph was collected from five larvae per sample during 5th instar and from two larvae per sample during 6th instar by cutting prolegs, and centrifuged at 500 × *g* for 5 min. Supernatants (50 µl) were used as haemolymph samples for SDS-PAGE and Western blotting. The pellets were washed with 100 µl phosphate-buffered saline by centrifugation and used as haemocyte samples. The RNAs (100 ng) were converted to cDNAs with SuperScript IV VILO MasterMix with ezDNase enzyme (Thermo Fisher Scientific). Half of the DNase-treated RNA sample was subjected to either reverse transcription (RT) or no-RT reaction following the manufacturer’s instructions. RT-PCRs for the MySEV SAM-dependent MTase gene were performed utilizing the CFX96 Real-Time PCR System (Bio-Rad). The β-actin gene (GenBank accession number ACX37085) of *M. separata* was used as an internal control. RT-PCRs targeting the β-actin gene using no-RT reactions as templates ascertained no DNA contamination. Primers used were 5′-GCATCTAACATAACCAATAATGACG-3′ and 5′-CTTTAACCCACGGTATACAAAAGAA-3′ for the MySEV SAM-dependent MTase gene, and 5′-GCCGCGACCTCACAGACTAC-3′ and 5′-CGAGAGCGACGTAGCAGAGC-3′ for the β-actin gene. Amplification reactions (10 µl) included 1 µl of 5-fold-diluted cDNAs, 5 µl THUNDERBIRD SYBR qPCR MIX (Toyobo), and 0.3 µM primers. The PCR conditions were 95 °C for 1 min, 40 cycles of 95 °C for 15 s and 60 °C for 30 s, and a melting curve analysis was then performed with sequential steps of 0.5 °C from 65 °C to 95 °C. A no- template control was also included in each run. Amplification of the target genes was confirmed by determining the specific melting temperatures of the products (72.5–73.0 °C for the MySEV SAM-dependent MTase gene, 85.0–85.5 °C for the β-actin gene). Sequence analyses in preliminary experiments further confirmed the amplification of targets.

Haemolymph samples were mixed with the same volume of 2× Laemmli buffer and boiled for 5 min, and 5 µl of these SDS-PAGE samples (corresponding to 2.5 µl of haemolymph) was electrophoresed and processed for Western blotting analysis as described above.

### Expression of host *JHAMT* in mock- and MySEV-infected insects

MySEV-infected and mock-infected *M. separata* larvae were prepared as above. Fat body, haemocyte, gut, Malpighian tubule, and epidermis samples were collected at 24-h intervals from 1 day after moulting to 5th instar to day 2 of 6th instar and analysed for *M. separata JHAMT* expression by RT-PCR. Fat bodies and haemocytes were collected as described above. Guts, Malpighian tubules, and epidermis were dissected from two or three larvae per sample. Three samples per tissue were analysed throughout the experiment. Total RNAs were extracted and subjected to DNase treatment and RT or no-RT reactions, and specific PCR for the *M. separata* JHAMT gene (GenBank accession number KM926339) was performed, all as described above. The β-actin gene was used as an internal control for RT-PCR. The *M. separata JHAMT* was amplified by the primers 5′-GTGCTGGCTCGCAACAACAA-3′ and 5′-AGTCGTGATAGGGCGACACGTA-3′. cDNA prepared from head tissues containing the corpora allata, where JH is synthesized and secreted, of day 2 of 5th instar *M. separata* was used as a positive-control template for *M. separata JHAMT* expression. A no-template control was also included in each run. Amplification of the target genes was confirmed by determining the specific melting temperatures of the products (82.5–83 °C for *M. separata JHAMT*, 85.0–85.5 °C for the β-actin gene). Sequence analyses of PCRs for *M. separata JHAMT* in preliminary experiments also confirmed the amplification of the target.

### Quantitative analysis of host JHAMT gene expression in head tissues of mock- and MySEV-infected insects

Quantitative RT-PCRs were performed for *M. separata JHAMT* expression in head tissues. MySEV-infected and mock-infected *M. separata* larvae were prepared as above, and heads were collected at 24-h intervals from 1 day after moulting to 5th instar to day 1 of 6th instar. Five samples were analysed for each time point. RNA extraction and cDNA sample preparation were conducted as described above. The absence of DNA contamination was also confirmed as above except for RT-PCRs targeting the RpL32 gene (GenBank accession number AB669190) (see Supplementary Fig. S3 for the primer sequences). *RpL32* was used as an internal control for normalization of expression levels (see Supplementary Method and Supplementary Fig. S3 for validation of *RpL32* as an internal control for expression normalization). Expression of the genes was measured in duplicate for each sample. Three no-template controls and a duplicate 5-fold dilution series of pooled cDNAs prepared from head tissues of day 2 of 5th instar mock-infected and EPV-infected larvae as standards were included in each run. Amplification reactions and PCR conditions were the same as described above. Amplification of the target genes was confirmed by determining the specific melting temperatures of the products (82.5–83 °C for *M. separata JHAMT*, 83.0–83.5 °C for *RpL32*). Relative normalized expression levels were calculated by the Pfaffl method^39^.

Data for the normalized expression levels were skewed to the right and fitted to lognormal distribution (Kolmogorov-Smirnov test, D = 0.0977, *p* = 0.1500). We thus logarithmically transformed the data and analysed them by generalized linear modelling techniques with normal error structure and identity link using JMP version 12 (SAS Institute). Days in instar, infection status (mock infection vs. EPV infection), and the interactions as explanatory variables were fitted to the data, and significant terms were determined. Insect *JHAMT* expression levels were generally downregulated in the final instar (i.e., 6th instar for *M. separata* larvae). Therefore, we compared the expression levels in 6th instar with those in 5th instar using contrast.

### Phylogenetic analysis

To construct phylogenetic trees, proteins with significant similarities as determined by alignment scores were extracted from the BLAST result for the MySEV SAM-dependent MTase, and aligned using MUSCLE incorporated in MEGA 7^40^. The resulting alignment was further checked using Gblocks^41^ to remove ambiguously aligned regions. Sequences with more than 90% identity with other sequences in the alignment, except for those in betaentomopoxviruses, were removed to decrease redundancy. Phylogenetic relationships among the proteins were inferred using the maximum likelihood method implemented in the RAxML BlackBox web server^42^. The appropriate model for protein evolution, i.e., LG with a gamma-distribution model of among-site rate heterogeneity and a proportion of invariant sites, was selected by ModelGenerator^43^.

### Data availability

The data supporting the findings of this study are included in this published article and its Supplementary Information file.

## Electronic supplementary material

Supplementary information

## References

[1] Ma K-W, Ma W (2016). Phytohormone pathways as targets of pathogens to facilitate infection. Plant. Mol. Biol..

[2] Steeg LG, Klein SL (2016). Sex steroids mediate bidirectional interactions between hosts and microbes. Horm. Behav..

[3] Regan JC (2013). Steroid hormone signaling is essential to regulate innate immune cells and fight bacterial infection in *Drosophila*. PLoS Path..

[4] Schwenke RA, Lazzaro BP (2017). Juvenile hormone suppresses resistance to infection in mated female *Drosophila melanogaster*. Curr. Biol..

[5] Beckage, N. E. *Parasites and pathogens: effects on host hormones and behavior*. (Chapman and Hall, 1997).

[6] Jindra M, Palli SR, Riddiford LM (2013). The juvenile hormone signaling pathway in insect development. Annu. Rev. Entomol..

[7] Yamanaka N, Rewitz KF, O’Connor MB (2013). Ecdysone control of developmental transitions: lessons from *Drosophila* research. Annu. Rev. Entomol..

[8] Skinner, M. A. *et al*. In *Virus taxonomy: Ninth Report of the International Committee on Taxonomy of Viruses* (eds A. M. King, M. J. Adams, E. B. Carstens, & E. J. Lefkowitz) 291–309 (Elsevior, (2011).

[9] Takatsuka J, Okuno S, Ishii T, Nakai M, Kunimi Y (2010). Fitness-related traits of entomopoxviruses isolated from *Adoxophyes honmai* (Lepidoptera: Tortricidae) at three localities in Japan. J. Invertebr. Pathol..

[10] Nakai M (2016). Entomopoxvirus infection induces changes in both juvenile hormone and ecdysteroid levels in larval *Mythimna separata*. J. Gen. Virol..

[11] Palli SR (2000). Choristoneura fumiferana entomopoxvirus prevents metamorphosis and modulates juvenile hormone and ecdysteroid titers. Insect Biochem. Mol. Biol..

[12] O’Reilly D, Miller L (1989). A baculovirus blocks insect molting by producing ecdysteroid UDP-glucosyl transferase. Science.

[13] Kiuchi M, Yasui H, Hayasaka S, Kamimura M (2003). Entomogenous fungus *Nomuraea rileyi* inhibits host insect molting by C22-oxidizing inactivation of hemolymph ecdysteroids. Arch. Insect Biochem. Physiol..

[14] Martin JL, McMillan FM (2002). SAM (dependent) I AM: the S-adenosylmethionine-dependent methyltransferase fold. Curr. Opin. Struct. Biol..

[15] Seman-Kamarulzaman A-F, Mohamed-Hussein Z-A, Ng CL, Hassan M (2016). Novel NAD^+^-farnesal dehydrogenase from *Polygonum minus* leaves. Purification and characterization of enzyme in juvenile hormone III biosynthetic pathway in plant. PLoS ONE.

[16] Yang Y (2006). An *Arabidopsis thaliana* methyltransferase capable of methylating farnesoic acid. Arch. Biochem. Biophys..

[17] Shinoda T, Itoyama K (2003). Juvenile hormone acid methyltransferase: a key regulatory enzyme for insect metamorphosis. Proc. Natl. Acad. Sci. USA.

[18] Chinchar VG (2017). ICTV Virus Taxonomy Profile: Iridoviridae. J. Gen. Virol..

[19] Mediannikov O, Sekeyova Z, Birg ML, Raoult D (2010). A novel obligate intracellular gamma-proteobacterium associated with ixodid ticks, *Diplorickettsia massiliensis*, Gen. Nov., Sp. Nov. PLoS One.

[20] Subramanian G (2012). Diplorickettsia massiliensis as a human pathogen. Eur. J. Clin. Microbiol. Infect. Dis..

[21] Sparagana SP, Bhaskaran G, Barrera P (1985). Juvenile hormone acid methyltransferase activity in imaginal discs of *Manduca sexta* prepupae. Arch. Insect. Biochem. Physiol..

[22] Ono M, Fukaya M (1969). The Juvenile-hormone-like effect of Chilo Iridescent Virus (CIV) on the metamorphosis of the silkworm, *Bombyx mori* L.: Lepidoptera: Bombycidae. Appl. Entomol. Zool..

[23] Ono M, Yagi S, Fukaya M (1972). Infectivity of Chilo iridescent virus against silkworm. Bull. Imperial Sericul. Exp. Stn..

[24] Ishii Y, Matsuura Y, Kakizawa S, Nikoh N, Fukatsu T (2013). Diversity of bacterial endosymbionts associated with macrosteles leafhoppers vectoring phytopathogenic phytoplasmas. Appl. Environ. Microbiol..

[25] Li K (2016). Diversity of bacteriome associated with *Phlebotomus chinensis* (Diptera: Psychodidae) sand flies in two wild populations from China. Sci. Rep..

[26] Miyakawa H, Toyota K, Sumiya E, Iguchi T (2014). Comparison of JH signaling in insects and crustaceans. Curr. Opin. Insect Sci..

[27] Nielsen MT (2013). *Aspergillus nidulans* synthesize insect juvenile hormones upon expression of a heterologous regulatory protein and in response to grazing by *Drosophila melanogaster* Larvae. PLoS ONE.

[28] Ares AM (2012). Liquid chromatography coupled to ion trap-tandem mass spectrometry to evaluate juvenile hormone III levels in bee hemolymph from *Nosema spp*. infected colonies. J. Chromatogr. B Analyt. Technol. Biomed. Life Sci..

[29] Down RE (2008). Infection by the microsporidium *Vairimorpha necatrix* (Microspora: Microsporidia) elevates juvenile hormone titres in larvae of the tomato moth, *Lacanobia oleracea* (Lepidoptera: Noctuidae). J. Invertebr. Pathol..

[30] Goblirsch M, Huang ZY, Spivak M (2013). Physiological and behavioral changes in honey bees (*Apis mellifera*) induced by *Nosema ceranae* infection. PLoS ONE.

[31] Fisher FM, Sanborn RC (1962). Production of insect juvenile hormone by the microsporidian parasite *Nosema*. Nature.

[32] Fisher FM, Sanborn RC (1964). *Nosema* as a source of juvenile hormone in parasitized insects. Biol. Bull..

[33] Cole TJ, Beckage NE, Tan FF, Srinivasan A, Ramaswamy SB (2002). Parasitoid-host endocrine relations: self-reliance or co-optation?. Insect Biochem. Mol. Biol..

[34] Romano MC, Jiménez P, Miranda-Brito C, Valdez RA (2015). Parasites and steroid hormones: corticosteroid and sex steroid synthesis, their role in the parasite physiology and development. Front. Neurosci..

[35] Thézé J, Takatsuka J, Nakai M, Arif B, Herniou E (2015). Gene acquisition convergence between entomopoxviruses and baculoviruses. Viruses.

[36] Hattori M, Atsusawa S (1980). Mass-rearing of the cabbage armyworm, *Mamestra brassicae* LINNE and the common armyworm, *Mythimna separata* WALKER (Lepidoptera: Noctuidae) on a simple artificial diet. Jap. J. Appl. Entomol. Zool..

[37] Hukuhara T, Xu JH, Yano K (1990). Replication of an entomopoxvirus in two lepidopteran cell lines. J. Invertebr. Pathol..

[38] Thézé J (2013). New insights into the evolution of *Entomopoxvirinae* from the complete genome sequences of four entomopoxviruses infecting *Adoxophyes honmai*, *Choristoneura biennis*, *Choristoneura rosaceana*, and *Mythimna separata*. J. Virol..

[39] Pfaffl MW (2001). A new mathematical model for relative quantification in real-time RT–PCR. Nucleic Acids Res..

[40] Kumar S, Stecher G, Tamura K (2016). MEGA7: Molecular Evolutionary Genetics Analysis Version 7.0 for bigger datasets. Mol. Biol. Evol..

[41] Castresana J (2000). Selection of conserved blocks from multiple alignments for their use in phylogenetic analysis. Mol. Biol. Evol..

[42] Stamatakis A, Hoover P, Rougemont J (2008). A rapid bootstrap algorithm for the RAxML Web servers. Syst. Biol..

[43] Keane TM, Creevey CJ, Pentony MM, Naughton TJ, Mclnerney JO (2006). Assessment of methods for amino acid matrix selection and their use on empirical data shows that ad hoc assumptions for choice of matrix are not justified. BMC Evol. Biol..

