# Supplementary material for: A virus carries a gene encoding juvenile hormone acid methyltransferase, a key regulatory enzyme in insect metamorphosis
Source: Sci Rep. 2017 Oct 19;7:13522. doi: 10.1038/s41598-017-14059-8 (PMC5648886; doi:10.1038/s41598-017-14059-8)
Supplement: Supplementary file 1 — Supplementary information [file 41598_2017_14059_MOESM1_ESM.pdf]

Supplementary information

This file contains Supplementary Table S1, Supplementary Figure S1, Supplementary Figure S2, Supplementary Figure S3, Supplementary Figure S4 and Supplementary Method.

A virus carries a gene encoding juvenile hormone acid methyltransferase, a key regulatory enzyme in insect metamorphosis

Jun Takatsuka[1,*], Madoka Nakai[2], and Tetsuro Shinoda[3]

[1]Forestry and Forest Products Research Institute, Forest Research and Management Organization, Tsukuba, Ibaraki, Japan

[2]Institute of Agriculture, Tokyo University of Agriculture and Technology, Fuchu, Tokyo, Japan

[3]Institute of Agrobiological Sciences, National Agriculture and Food Research Organization, Tsukuba, Ibaraki, Japan

*Address correspondence to Jun Takatsuka, junsan@ffpri.affrc.go.jp.

Supplementary Table S1. Part of BLASTP search results of Mythimna separata entomopoxvirus S-adenosyl-L-methionine dependent methyltransferase

Sequence	Bit score	Definition	% identity	E-value	Organism
YP_008003556.1	534	similar_to_AMEV004_Mythimna_separata_entomopoxvirus_L	100	0	Virus
YP_008004135.1	309	similar_to_AMEV004_Choristoneura_biennis_entomopoxvirus	62	8.94E-103	Virus
YP_008004449.1	308	similar_to_AMEV004_Choristoneura_rosaceana_entomopoxvirus_L	63	2.36E-102	Virus
NP_064786.1	290	SAM-dependent_methyltransferase_Amsacta_moorei_entomopoxvirus	59	1.77E-95	Virus
YP_008003847.1	273	similar_to_AMEV004_Adoxophyes_honmai_entomopoxvirus_L	55	1.43E-88	Virus
NP_149698.1	237	235L_Invertebrate_iridescent_virus_6	44	1.55E-74	Virus
WP_010597711.1	171	SAM-dependent_methyltransferase_Diplorickettsia_massiliensis	34	6.14E-49	Bacterium
WP_019233023.1	132	MULTISPECIES:_SAM-dependent_methyltransferase_Legionella	29	1.07E-33	Bacterium
OGV27197.1	131	hypothetical_protein_A3F18_07225_Legionellales_bacterium_RIFCSPHIGHO2_12_FULL_37_14	31	1.86E-33	Bacterium
WP_058387236.1	130	class_I_SAM-dependent_methyltransferase_Legionella_cherrii	31	7.34E-33	Bacterium
OGT41298.1	130	hypothetical_protein_A3F13_09880_Gammaproteobacteria_bacterium_RIFCSPHIGHO2_12_FULL_40_19	30	9.32E-33	Bacterium
WP_012187506.1	129	MULTISPECIES:_SAM-dependent_methyltransferase_Fluoribacter	30	1.54E-32	Bacterium
WP_058450543.1	128	SAM-dependent_methyltransferase_Legionella_jamestowniensis	29	6.22E-32	Bacterium
OGT53634.1	124	hypothetical_protein_A3F17_01115_Gammaproteobacteria_bacterium_RIFCSPHIGHO2_12_FULL_41_15	29	1.00E-30	Bacterium
WP_045105130.1	124	class_I_SAM-dependent_methyltransferase_Legionella_hackeliae	28	2.45E-30	Bacterium
WP_083503742.1	122	class_I_SAM-dependent_methyltransferase_Legionella_nautarum	29	6.09E-30	Bacterium
OJU81647.1	121	hypothetical_protein_BGO10_08585_Chlamydia_sp._32-24	30	2.67E-29	Bacterium
WP_026387652.1	117	class_I_SAM-dependent_methyltransferase_Acidobacteria_bacterium_KBS_146	28	4.58E-28	Bacterium
OAI52787.1	117	methyltransferase_type_11_Planctomycetaceae_bacterium_SCGC_AG-212-F19	27	5.66E-28	Bacterium
OQX00207.1	117	hypothetical_protein_BWK74_00235_Desulfobacteraceae_bacterium_A6	24	8.01E-28	Bacterium
WP_058527768.1	116	class_I_SAM-dependent_methyltransferase_Legionella_erythra	28	9.59E-28	Bacterium
WP_035347762.1	116	SAM-dependent_methyltransferase_Edaphobacter_aggregans	28	1.18E-27	Bacterium
WP_084758898.1	115	class_I_SAM-dependent_methyltransferase_Legionella_adelaidensis	29	2.73E-27	Bacterium
WP_068280870.1	115	SAM-dependent_methyltransferase_Rhodococcus_kunmingensis	26	3.38E-27	Bacterium
WP_058445789.1	114	SAM-dependent_methyltransferase_Legionella_feeleii	29	1.56E-26	Bacterium
WP_052358047.1	113	SAM-dependent_methyltransferase_Legionella_pneumophila	27	2.32E-26	Bacterium
WP_020711554.1	112	hypothetical_protein_Acidobacteriaceae_bacterium_KBS_83	28	2.48E-26	Bacterium
WP_020412182.1	112	class_I_SAM-dependent_methyltransferase_Microbulbifer_variabilis	27	2.90E-26	Bacterium
WP_010844925.1	111	class_I_SAM-dependent_methyltransferase_Gordonia_terrae	27	1.36E-25	Bacterium
WP_087537581.1	111	hypothetical_protein_Nostocales_cyanobacterium_HT-58-2	28	1.36E-25	Bacterium
ODT67862.1	110	hypothetical_protein_ABS71_10275_bacterium_SCN_62-11	25	2.18E-25	Bacterium
OGP52632.1	109	SAM-dependent_methyltransferase_Deltaproteobacteria_bacterium_RBG_13_52_11	27	6.06E-25	Bacterium
WP_064280382.1	108	SAM-dependent_methyltransferase_Mycobacterium_iranicum	23	6.22E-25	Bacterium
KPK10886.1	108	SAM-dependent_methyltransferase_Anaerolineae_bacterium_SG8_19	25	1.26E-24	Bacterium
WP_012627509.1	107	class_I_SAM-dependent_methyltransferase_Cyanothece_sp._PCC_7425	26	2.41E-24	Bacterium
WP_081776777.1	107	hypothetical_protein_Legionella_geestiana	25	4.06E-24	Bacterium
WP_058452204.1	107	SAM-dependent_methyltransferase_Legionella_maceachernii	27	4.32E-24	Bacterium
ABK15288.1	107	Methyltransferase_type_11_Methanosaeta_thermophila_PT	25	4.90E-24	Archaeon
OGN56528.1	107	hypothetical_protein_A2796_07270_Chlamydiae_bacterium_RIFCSPHIGHO2_01_FULL_44_39	26	9.35E-24	Bacterium
WP_055475956.1	106	class_I_SAM-dependent_methyltransferase_Gordonia_sp._HS-NH1	26	9.35E-24	Bacterium
EKE08103.1	107	hypothetical_protein_ACD_17C00346G0002_partial_uncultured_bacterium	26	9.72E-24	Bacterium
WP_087538176.1	105	SAM-dependent_methyltransferase_Nostocales_cyanobacterium_HT-58-2	27	1.20E-23	Bacterium
WP_011021804.1	105	SAM-dependent_methyltransferase_Methanosarcina_acetivorans	27	1.52E-23	Archaeon
WP_007313851.1	105	SAM-dependent_methyltransferase_Methanolinea_tarda	27	1.62E-23	Bacterium
OGN55150.1	105	hypothetical_protein_A2098_04020_Chlamydiae_bacterium_GWF2_49_8	27	1.83E-23	Bacterium
KIA78318.1	105	hypothetical_protein_DB43_EH00070_Parachlamydia_acanthamoebae	28	1.97E-23	Bacterium
OGN53132.1	105	hypothetical_protein_A2Z85_02730_Chlamydiae_bacterium_GWA2_50_15	27	2.30E-23	Bacterium
WP_011597893.1	104	SAM-dependent_methyltransferase_Rhodococcus_jostii	25	2.89E-23	Bacterium
WP_013718747.1	105	SAM-dependent_methyltransferase_Methanosaeta_concilii	27	3.11E-23	Archaeon
WP_006341661.1	104	hypothetical_protein_Parachlamydia_acanthamoebae	29	3.72E-23	Bacterium
WP_059359134.1	104	hypothetical_protein_Parachlamydia_acanthamoebae	29	4.26E-23	Bacterium
WP_016883413.1	104	SAM-dependent_methyltransferase_Rhodococcus_sp._DK17	25	4.29E-23	Bacterium
OGV46270.1	104	SAM-dependent_methyltransferase_Lentisphaerae_bacterium_GWF2_57_35	26	4.79E-23	Bacterium
WP_013644788.1	103	SAM-dependent_methyltransferase_Methanobacterium_lacus	28	6.91E-23	Archaeon
WP_014928579.1	103	SAM-dependent_methyltransferase_Gordonia_sp._KTR9	26	8.68E-23	Bacterium
OGN70059.1	103	hypothetical_protein_A3I15_02110_Chlamydiae_bacterium_RIFCSPLOWO2_02_FULL_49_12	27	8.95E-23	Bacterium
WP_051551075.1	103	class_I_SAM-dependent_methyltransferase_Legionella_geestiana	25	1.13E-22	Bacterium
WP_036252469.1	103	SAM-dependent_methyltransferase_Methylobacter_sp._BBA5.1	27	1.19E-22	Bacterium
WP_071105437.1	103	SAM-dependent_methyltransferase_Moorea_producens	28	1.37E-22	Bacterium
WP_053063426.1	103	SAM-dependent_methyltransferase_Piscirickettsia_salmonis	31	1.39E-22	Bacterium
WP_009479026.1	102	SAM-dependent_methyltransferase_Rhodococcus_sp._JVH1	25	1.41E-22	Bacterium
WP_007529070.1	102	class_I_SAM-dependent_methyltransferase_Rhodococcus_triatomae	24	1.76E-22	Bacterium
WP_022847427.1	102	SAM-dependent_methyltransferase_Desulfurobacterium_sp._TC5-1	28	1.85E-22	Bacterium
WP_013818761.1	102	type_11_methyltransferase_Methylomonas_methanica	27	1.93E-22	Bacterium
WP_023175350.1	101	class_I_SAM-dependent_methyltransferase_Gloeobacter_kilaueensis	26	4.68E-22	Bacterium
WP_087910684.1	101	hypothetical_protein_Piscirickettsia_salmonis	30	4.82E-22	Bacterium
WP_019050011.1	101	MULTISPECIES:_class_I_SAM-dependent_methyltransferase_Actinobacteria	24	4.85E-22	Bacterium
WP_061700313.1	101	SAM-dependent_methyltransferase_Rhodococcus_sp._LB1	24	5.60E-22	Bacterium
WP_031937253.1	101	SAM-dependent_methyltransferase_Rhodococcus_defluvii	21	6.10E-22	Bacterium
AII05270.1	101	SAM-dependent_methyltransferase_Rhodococcus_opacus	23	6.22E-22	Bacterium
KQC16415.1	101	SAM-dependent_methyltransferase_Methanosaeta_sp._SDB	23	6.38E-22	Archaeon
WP_048119776.1	101	SAM-dependent_methyltransferase_Methanosarcina_vacuolata	25	6.52E-22	Archaeon
WP_078112569.1	101	SAM-dependent_methyltransferase_Gordonia_sp._IITR100	26	7.77E-22	Bacterium
WP_020505314.1	101	class_I_SAM-dependent_methyltransferase_Lamprocystis_purpurea	23	7.96E-22	Bacterium
WP_058252819.1	100	SAM-dependent_methyltransferase_Gordonia_sp._SGD-V-85	26	8.26E-22	Bacterium
WP_032125542.1	101	MULTISPECIES:_hypothetical_protein_Chlamydiia	28	8.29E-22	Bacterium
WP_020160969.1	100	hypothetical_protein_Methylobacter_marinus	26	9.45E-22	Bacterium

WP_048154704.1	100	class_I_SAM-dependent_methyltransferase_Methanosarcina_sp._Kolksee	25	9.77E-22	Archaeon
WP_036298287.1	100	SAM-dependent_methyltransferase_Methylobacter_whittenburyi	26	9.84E-22	Bacterium
OGC22310.1	100	SAM-dependent_methyltransferase_candidate_division_WOR-1_bacterium_RIFOXYA2_FULL_41_14	27	9.93E-22	Bacterium
WP_067452987.1	100	SAM-dependent_methyltransferase_Nocardia_alba	22	1.04E-21	Bacterium
ANH78853.1	101	Methyltransferase_type_11_Chlamydia_sp._2742-308	26	1.27E-21	Bacterium
WP_075900116.1	100	SAM-dependent_methyltransferase_Moorea_bouillonii	26	1.36E-21	Bacterium
WP_027159946.1	100	SAM-dependent_methyltransferase_Methylobacter_luteus	28	1.72E-21	Bacterium
WP_048122647.1	100	MULTISPECIES:_SAM-dependent_methyltransferase_Methanosarcina	26	1.92E-21	Archaeon
WP_082905013.1	100	hypothetical_protein_Chlamydia_sp._2742-308	26	2.04E-21	Bacterium
AKB55347.1	100	Trans-aconitate_2-methyltransferase_Methanosarcina_barkeri_MS	26	2.12E-21	Archaeon
WP_005238856.1	99.8	SAM-dependent_methyltransferase_Rhodococcus_opacus	24	2.37E-21	Bacterium
WP_061041483.1	99.8	SAM-dependent_methyltransferase_Rhodococcus_sp._SC4	24	2.45E-21	Bacterium
WP_084839582.1	99.8	SAM-dependent_methyltransferase_Williamsia_sp._1138	23	2.71E-21	Bacterium
WP_065629464.1	99.4	SAM-dependent_methyltransferase_Gordonia_sp._UCD-TK1	25	3.18E-21	Bacterium
WP_004020473.1	99	SAM-dependent_methyltransferase_Gordonia_terrae	25	4.56E-21	Bacterium
WP_087560228.1	99	SAM-dependent_methyltransferase_Rhodococcus_sp._NCIMB_12038	22	4.95E-21	Bacterium
WP_020522886.1	98.6	SAM-dependent_methyltransferase_Catelliglobosispora_koreensis	24	5.49E-21	Bacterium
WP_064571697.1	99	SAM-dependent_methyltransferase_Gordonia_sp._LAM0048	25	5.65E-21	Bacterium
WP_069311842.1	98.6	class_I_SAM-dependent_methyltransferase_Piscirickettsia_litoralis	29	5.94E-21	Bacterium
WP_058996591.1	98.6	SAM-dependent_methyltransferase_Leptolyngbya_sp._NIES-2104	29	6.03E-21	Bacterium
KQC15905.1	98.6	SAM-dependent_methyltransferase_partial_Methanosaeta_sp._SDB	23	6.11E-21	Archaeon
WP_072940888.1	98.6	class_I_SAM-dependent_methyltransferase_Rhodococcus_koreensis	23	6.89E-21	Bacterium
WP_056819464.1	98.6	SAM-dependent_methyltransferase_Nocardia_sp._Root136	22	7.21E-21	Bacterium
WP_018162930.1	98.2	SAM-dependent_methyltransferase_Smaragdicoccus_niigatensis	22	7.67E-21	Bacterium
KUK44115.1	98.2	Methyltransferase_putative_Methanosaeta_harundinacea	23	8.66E-21	Archaeon
KHD04924.1	98.2	SAM-dependent_methyltransferase_Candidatus_Thiomargarita_nelsonii	28	8.77E-21	Bacterium
WP_017364724.1	98.2	SAM-dependent_methyltransferase_Methylococcus_capsulatus	26	9.50E-21	Bacterium
WP_050063296.1	97.8	SAM-dependent_methyltransferase_Rhodococcus_sp._RD6.2	23	1.12E-20	Bacterium
OFW57920.1	98.2	SAM-dependent_methyltransferase_Actinobacteria_bacterium_RBG_13_55_18	23	1.22E-20	Bacterium
WP_020481712.1	97.8	hypothetical_protein_Methylomonas_sp._MK1	26	1.37E-20	Bacterium
XP_728723.1	97.4	hypothetical_protein_partial_Plasmodium_yoelii_yoelii_17XNL	25	1.51E-20	Bacterium
WP_059022185.1	97.4	SAM-dependent_methyltransferase_Mycobacterium_sp._M26	23	1.57E-20	Bacterium
WP_068970507.1	97.4	SAM-dependent_methyltransferase_Nocardia_farcinica	25	1.79E-20	Bacterium
OJV39771.1	97.4	methyltransferase_type_11_Acidobacteriales_bacterium_59-55	26	1.99E-20	Bacterium
WP_086536096.1	97.1	SAM-dependent_methyltransferase_Gordonia_lacunae	24	2.11E-20	Bacterium
WP_010960300.1	97.1	SAM-dependent_methyltransferase_Methylococcus_capsulatus	25	2.26E-20	Bacterium
WP_015126211.1	97.1	class_I_SAM-dependent_methyltransferase_Synechococcus_sp._PCC_6312	26	2.36E-20	Bacterium
WP_013906740.1	97.1	SAM-dependent_methyltransferase_Thermodesulfatator_indicus	25	2.41E-20	Bacterium
WP_072749933.1	97.1	SAM-dependent_methyltransferase_Rhodococcus_maanshanensis	22	2.50E-20	Bacterium
WP_015889586.1	97.1	class_I_SAM-dependent_methyltransferase_Rhodococcus_opacus	23	2.55E-20	Bacterium
WP_036276857.1	97.1	MULTISPECIES:_SAM-dependent_methyltransferase_Methylomonas	26	2.64E-20	Bacterium
WP_015748062.1	97.1	SAM-dependent_methyltransferase_Nakamurella_multipartita	23	2.75E-20	Bacterium
ESP62633.1	97.1	hypothetical_protein_SMITH_706_Smithella_sp._ME-1	30	2.88E-20	Bacterium
KJS80682.1	96.7	SAM-dependent_methyltransferase_Peptococcaceae_bacterium_BICA1-8	27	3.04E-20	Bacterium
WP_084893088.1	96.7	SAM-dependent_methyltransferase_Williamsia_sp._1135	23	3.67E-20	Bacterium
OGT32001.1	96.3	hypothetical_protein_A3E87_10645_Gammaproteobacteria_bacterium_RIFCSPHIGHO2_12_FULL_35_23	25	5.02E-20	Bacterium
OHX37336.1	96.3	SAM-dependent_methyltransferase_Methylomonas_sp._LWB	27	5.65E-20	Bacterium
WP_083385734.1	96.3	SAM-dependent_methyltransferase_Methylomonas_sp._LWB	27	6.82E-20	Bacterium
WP_082885465.1	96.3	hypothetical_protein_Methylomonas_koyamae	25	8.43E-20	Bacterium
WP_063054937.1	95.5	SAM-dependent_methyltransferase_Nocardia_soli	22	9.51E-20	Bacterium
KKP25082.1	95.1	Trans-aconitate_2-methyltransferase_candidate_division_TM6_bacterium_GW2011_GWE2_31_21	28	1.01E-19	Bacterium
WP_019866949.1	95.5	hypothetical_protein_Methylovulum_miyakonense	26	1.15E-19	Bacterium
WP_013862476.1	95.1	class_I_SAM-dependent_methyltransferase_Microlunatus_phosphovorus	23	1.20E-19	Bacterium
WP_058503462.1	95.1	SAM-dependent_methyltransferase_Legionella_nautarum	27	1.40E-19	Bacterium
WP_061818900.1	94.7	class_I_SAM-dependent_methyltransferase_Legionella_pneumophila	30	1.76E-19	Bacterium
WP_066580736.1	94.7	class_I_SAM-dependent_methyltransferase_Clostridium_sp._Marseille-P2538	26	1.78E-19	Bacterium
WP_062992424.1	94.7	SAM-dependent_methyltransferase_Nocardia_salmonicida	22	1.89E-19	Bacterium
WP_011308538.1	94.4	class_I_SAM-dependent_methyltransferase_Methanosarcina_barkeri	26	2.26E-19	Archaeon
WP_029633625.1	93.6	hypothetical_protein_Scytonema_hofmanni_UTEX_B_1581	32	4.23E-19	Bacterium
WP_082879758.1	93.6	SAM-dependent_methyltransferase_Methylomonas_methanica	26	5.46E-19	Bacterium
KQC03571.1	93.6	SAM-dependent_methyltransferase_Methanoculleus_sp._SDB	22	5.82E-19	Archaeon
WP_053331853.1	93.6	SAM-dependent_methyltransferase_Criblamydia_sequanensis	24	7.07E-19	Bacterium
WP_078771972.1	92.8	MULTISPECIES:_trans-aconitate_methyltransferase_Elizabethkingia	26	7.12E-19	Bacterium
WP_063003181.1	92.8	SAM-dependent_methyltransferase_Nocardia_cummidelens	23	8.32E-19	Bacterium
OJV47827.1	93.6	hypothetical_protein_BGO28_05845_Alphaproteobacteria_bacterium_43-37	29	8.37E-19	Bacterium
OEU42239.1	92.8	SAM-dependent_methyltransferase_Methanosarcina_sp._Ant1	25	1.06E-18	Archaeon
WP_054300328.1	92.4	hypothetical_protein_Piscirickettsia_salmonis	29	1.23E-18	Bacterium
WP_086133626.1	92	SAM-dependent_methyltransferase_Methylocaldum_sp._SAD2	23	1.58E-18	Bacterium
WP_065081115.1	92	trans-aconitate_methyltransferase_Elizabethkingia_miricola	27	1.60E-18	Bacterium
WP_078795875.1	92	trans-aconitate_methyltransferase_Elizabethkingia_miricola	27	1.76E-18	Bacterium
WP_049037311.1	91.7	trans-aconitate_methyltransferase_Elizabethkingia_anophelis	27	1.93E-18	Bacterium
WP_078674744.1	91.7	trans-aconitate_methyltransferase_Elizabethkingia_anophelis	27	1.95E-18	Bacterium
WP_082304435.1	90.1	hypothetical_protein_Piscirickettsia_salmonis	31	1.99E-18	Bacterium
WP_054701608.1	92	hypothetical_protein_Desulfosarcina_cetonica	24	2.17E-18	Bacterium
WP_058466413.1	91.7	class_I_SAM-dependent_methyltransferase_Legionella_cincinnatiensis	28	2.17E-18	Bacterium
WP_078780979.1	91.7	trans-aconitate_methyltransferase_Elizabethkingia_miricola	27	2.25E-18	Bacterium
WP_035590265.1	91.7	trans-aconitate_methyltransferase_Elizabethkingia_anophelis	27	2.42E-18	Bacterium
WP_009085676.1	91.7	trans-aconitate_2-methyltransferase_Elizabethkingia_anophelis	27	2.55E-18	Bacterium
WP_034848261.1	91.3	MULTISPECIES:_trans-aconitate_methyltransferase_Elizabethkingia	27	2.74E-18	Bacterium

XP_011141693.1	92.4	juvenile_hormone_acid_O-methyltransferase_Harpegnathos_saltator	29	2.79E-18	Eukaryote(Insect)
XP_002602352.1	91.7	hypothetical_protein_BRAFLDRAFT_98019_Branchiostoma_floridae	25	2.87E-18	Eukaryote(Amphioxus)
WP_078406811.1	91.3	trans-aconitate_methyltransferase_Elizabethkingia_endophytica	27	2.94E-18	Bacterium
AQW98094.1	91.3	trans-aconitate_methyltransferase_Elizabethkingia_anophelis	27	2.97E-18	Bacterium
WP_087093635.1	91.3	trans-aconitate_methyltransferase_Elizabethkingia_anophelis	27	3.29E-18	Bacterium
WP_059156762.1	90.9	MULTISPECIES:_trans-aconitate_methyltransferase_Elizabethkingia	27	3.80E-18	Bacterium
WP_061637413.1	90.9	class_I_SAM-dependent_methyltransferase_Legionella_pneumophila	29	4.36E-18	Bacterium
WP_086982441.1	90.9	trans-aconitate_methyltransferase_Elizabethkingia_anophelis	27	4.62E-18	Bacterium
WP_020105852.1	90.9	class_I_SAM-dependent_methyltransferase_Nocardia_sp._348MFTsu5.1	25	5.46E-18	Bacterium
SDX46165.1	90.9	trans-aconitate_2-methyltransferase_Thiocapsa_roseopersicina	22	5.61E-18	Bacterium
KKQ11788.1	90.5	hypothetical_protein_US22_C0013G0002_candidate_division_TM6_bacterium_GW2011_GWF2_36_6	28	5.70E-18	Bacterium
WP_061890102.1	90.5	trans-aconitate_methyltransferase_Elizabethkingia_anophelis	27	6.54E-18	Bacterium
EFN83053.1	90.9	Uncharacterized_protein_yxbB_Harpegnathos_saltator	30	6.59E-18	Eukaryote(Insect)
WP_058877416.1	90.5	trans-aconitate_methyltransferase_Elizabethkingia_anophelis	27	6.75E-18	Bacterium
WP_012979142.1	90.1	class_I_SAM-dependent_methyltransferase_Legionella_longbeachae	29	7.97E-18	Bacterium
WP_078678402.1	89.7	trans-aconitate_methyltransferase_Elizabethkingia_genomosp._3	26	1.22E-17	Bacterium
WP_019505938.1	89.7	hypothetical_protein_Pleurocapsa_sp._PCC_7319	29	1.43E-17	Bacterium
WP_047033197.1	89.4	trans-aconitate_methyltransferase_Elizabethkingia_anophelis	27	1.56E-17	Bacterium
WP_077732800.1	89.4	SAM-dependent_methyltransferase_Methylocaldum_sp._14B	23	1.86E-17	Bacterium
WP_078703245.1	89	trans-aconitate_methyltransferase_Elizabethkingia_genomosp._3	26	2.06E-17	Bacterium
WP_059343529.1	89	MULTISPECIES:_trans-aconitate_methyltransferase_Elizabethkingia	26	2.08E-17	Bacterium
XP_014279174.1	89.4	juvenile_hormone_acid_O-methyltransferase-like_Halyomorpha_halys	26	2.28E-17	Eukaryote(Insect)
WP_045227215.1	89	SAM-dependent_methyltransferase_Methylococcaceae_bacterium_73a	26	2.45E-17	Bacterium
WP_045182227.1	88.6	trans-aconitate_methyltransferase_Elizabethkingia_miricola	26	2.60E-17	Bacterium
KFN38754.1	88.6	hypothetical_protein_JU82_10275_Sulfuricurvum_sp._MLSB	31	3.41E-17	Bacterium
CRI64269.1	89	Methyltransferase_type_11_Thiocapsa_sp._KS1	23	3.57E-17	Bacterium
OGV48550.1	88.6	SAM-dependent_methyltransferase_Lentisphaerae_bacterium_GWF2_52_8	23	3.85E-17	Bacterium
WP_014587257.1	88.6	SAM-dependent_methyltransferase_Methanosaeta_harundinacea	30	3.96E-17	Archaeon
WP_034869602.1	88.2	MULTISPECIES:_trans-aconitate_methyltransferase_Elizabethkingia	28	4.00E-17	Bacterium
WP_038351698.1	88.2	methyltransferase_type_11_Eubacterium_limosum	28	4.49E-17	Bacterium
WP_078722046.1	88.2	trans-aconitate_methyltransferase_Elizabethkingia_genomosp._4	25	4.57E-17	Bacterium
ALT10380.1	88.2	juvenile_hormone_acid_methyltransferase_Portunus_trituberculatus	24	5.33E-17	Eukaryote(Crab)
KHG32957.1	87.4	hypothetical_protein_OA34_12520_Sulfurospirillum_sp._MES	26	7.40E-17	Bacterium
WP_006566370.1	87.4	methyltransferase_type_11_Anaerostipes_caccae	27	9.78E-17	Archaeon
WP_082006818.1	87.4	hypothetical_protein_Sulfurospirillum_cavolei	28	1.09E-16	Bacterium
WP_068470177.1	87.8	hypothetical_protein_Parachlamydia_sp._C2	26	1.16E-16	Bacterium
WP_087438087.1	87	hypothetical_protein_Sulfurospirillum_sp._SL2-1	34	1.18E-16	Bacterium
WP_078778194.1	86.3	trans-aconitate_methyltransferase_Elizabethkingia_sp._C1558	25	2.11E-16	Bacterium
WP_082709180.1	86.3	hypothetical_protein_Sulfurospirillum_cavolei	29	2.93E-16	Bacterium
WP_078798862.1	85.5	trans-aconitate_methyltransferase_Elizabethkingia_miricola	25	3.61E-16	Bacterium
WP_048190665.1	85.9	SAM-dependent_methyltransferase_Methanobacterium_sp._SMA-27	26	3.70E-16	Archaeon
XP_017780260.1	85.9	juvenile_hormone_acid_O-methyltransferase-like_Nicrophorus_vespilloides	27	4.48E-16	Eukaryote(Insect)
WP_059326048.1	85.5	trans-aconitate_methyltransferase_Elizabethkingia_genomosp._4	25	4.52E-16	Bacterium
OGO01099.1	85.5	SAM-dependent_methyltransferase_Chloroflexi_bacterium_RBG_13_53_26	23	5.02E-16	Bacterium
WP_069477615.1	85.1	SAM-dependent_methyltransferase_Sulfurospirillum_halorespirans	26	5.83E-16	Bacterium
WP_041018469.1	85.1	SAM-dependent_methyltransferase_Criblamydia_sequanensis	28	5.95E-16	Bacterium
WP_051929550.1	82.8	SAM-dependent_methyltransferase_Piscirickettsia_salmonis	31	6.99E-16	Bacterium
WP_049728600.1	85.1	methyltransferase_type_11_Dorea_sp._D27	25	7.10E-16	Bacterium
XP_017772157.1	85.1	juvenile_hormone_acid_O-methyltransferase-like_Nicrophorus_vespilloides	29	7.62E-16	Eukaryote(Insect)
WP_078403375.1	84.7	trans-aconitate_methyltransferase_Elizabethkingia_genomosp._4	25	8.15E-16	Bacterium
WP_011844790.1	84.7	SAM-dependent_methyltransferase_Methanoculleus_marisnigri	27	8.61E-16	Archaeon
OGN61574.1	84.3	hypothetical_protein_A3F09_05900_Chlamydiae_bacterium_RIFCSPHIGHO2_12_FULL_49_11	27	1.00E-15	Bacterium
WP_073360163.1	84.3	class_I_SAM-dependent_methyltransferase_Rhodococcus_jostii	23	1.16E-15	Bacterium
WP_002979853.1	84.3	trans-aconitate_2-methyltransferase_Chryseobacterium_gleum	28	1.21E-15	Bacterium
ALI37693.1	84.3	Trans-aconitate_2-methyltransferase_Candidatus_Nitrocosmicus_oleophilus	26	1.52E-15	Bacterium
WP_067043100.1	83.6	class_I_SAM-dependent_methyltransferase_Moritella_sp._JT01	27	1.88E-15	Bacterium
WP_029914722.1	83.6	methyltransferase_type_11_Pelobacter_seleniigenes	31	2.12E-15	Bacterium
WP_034694292.1	83.6	trans-aconitate_methyltransferase_Chryseobacterium_sp._CF365	27	2.13E-15	Bacterium
KJH70650.1	83.6	SAM-dependent_methyltransferase_Aliterella_atlantica_CENA595	25	2.57E-15	Bacterium
WP_070904872.1	83.2	trans-aconitate_methyltransferase_Elizabethkingia_meningoseptica	26	3.37E-15	Bacterium
XP_019616241.1	83.2	juvenile_hormone_acid_O-methyltransferase-like_Branchiostoma_belcheri	24	3.40E-15	Eukaryote(Amphioxus)
WP_015757552.1	83.2	SAM-dependent_methyltransferase_Desulfotomaculum_acetoxidans	24	3.82E-15	Bacterium
WP_069215371.1	82.4	trans-aconitate_methyltransferase_Elizabethkingia_meningoseptica	26	5.25E-15	Bacterium
WP_023495483.1	82.8	methyltransferase_235L_Methyloglobulus_morosus	37	5.38E-15	Bacterium
WP_036745856.1	83.2	SAM-dependent_methyltransferase_Parachlamydia_acanthamoebae	24	5.50E-15	Bacterium
BAU79434.1	82.8	juvenile_hormone_acid_O-methyltransferase_Planococcus_kraunhiae	31	5.55E-15	Eukaryote(Insect)
WP_016198724.1	82.4	trans-aconitate_2-methyltransferase_Elizabethkingia_meningoseptica	26	5.81E-15	Bacterium
WP_047375168.1	82.4	trans-aconitate_methyltransferase_Chryseobacterium_sp._YR459	26	6.18E-15	Bacterium
EFB42723.1	83.2	hypothetical_protein_pah_c004o293_Parachlamydia_acanthamoebae_str._Halls_coccus	24	6.37E-15	Bacterium
WP_013925220.1	82.8	SAM-dependent_methyltransferase_Parachlamydia_acanthamoebae	24	7.13E-15	Bacterium
XP_019627372.1	82	juvenile_hormone_acid_O-methyltransferase-like_Branchiostoma_belcheri	21	8.09E-15	Eukaryote(Amphioxus)
XP_011501098.1	82	uncharacterized_protein_LOC105364772_isoform_X2_Ceratosolen_solmsi_marchali	30	9.79E-15	Eukaryote(Insect)
WP_064041233.1	82	SAM-dependent_methyltransferase_Methylomonas_koyamae	22	1.05E-14	Bacterium
WP_079241519.1	81.6	trans-aconitate_methyltransferase_Chryseobacterium_indologenes	27	1.07E-14	Bacterium
WP_076597375.1	81.3	trans-aconitate_methyltransferase_Chryseobacterium_sp._RU33C	28	1.46E-14	Bacterium
WP_058531577.1	81.3	SAM-dependent_methyltransferase_Legionella_rubrilucens	32	1.47E-14	Bacterium
SDF63959.1	81.3	Trans-aconitate_methyltransferase_Methanolobus_vulcani	24	1.66E-14	Archaeon
CDC36350.1	81.3	methyltransferase_domain_protein_Anaerostipes_sp._CAG:276	27	1.70E-14	Archaeon
CDZ80815.1	81.3	Trans-aconitate_2-methyltransferase_Candidatus_Rubidus_massiliensis	27	1.91E-14	Bacterium

WP_082065417.1	82.8	hypothetical_protein_Aliterella_atlantica	25	2.09E-14	Bacterium
WP_028894045.1	80.9	hypothetical_protein_Syntrophorhabdus_aromaticivorans	21	2.17E-14	Bacterium
XP_002610487.1	80.9	hypothetical_protein_BRAFLDRAFT_65649_Branchiostoma_floridae	21	2.24E-14	Eukaryote(Amphioxus)
WP_058507383.1	80.9	class_I_SAM-dependent_methyltransferase_Legionella_quinlivanii	27	2.27E-14	Bacterium
XP_002612666.1	81.6	hypothetical_protein_BRAFLDRAFT_78703_Branchiostoma_floridae	24	2.32E-14	Eukaryote(Amphioxus)
WP_027222839.1	80.9	SAM-dependent_methyltransferase_Legionella_pneumophila	28	2.35E-14	Bacterium
OHD78787.1	80.5	methyltransferase_type_11_partial_Spirochaetes_bacterium_RIFOXYB1_FULL_32_8	29	2.50E-14	Bacterium
OHD39969.1	80.5	methyltransferase_type_11_Spirochaetes_bacterium_GWE1_32_154	29	3.01E-14	Bacterium
WP_058694659.1	80.5	methyltransferase_type_11_Eubacterium_limosum	26	3.05E-14	Bacterium
AHZ20738.1	80.9	juvenile_hormone_acid_methyltransferase_Diploptera_punctata	24	3.05E-14	Eukaryote(Insect)
WP_073552195.1	80.5	trans-aconitate_methyltransferase_Elizabethkingia_meningoseptica	25	3.10E-14	Bacterium
ALU14753.1	80.5	SAM-dependent_methyltransferase_Eubacterium_limosum	26	3.16E-14	Bacterium
WP_028385907.1	80.5	class_I_SAM-dependent_methyltransferase_Legionella_geestiana	29	3.30E-14	Bacterium
XP_019628710.1	80.1	juvenile_hormone_acid_O-methyltransferase-like_Branchiostoma_belcheri	23	3.45E-14	Eukaryote(Amphioxus)
WP_061483798.1	80.1	class_I_SAM-dependent_methyltransferase_Legionella_pneumophila	28	4.33E-14	Bacterium

Green rows show insect S-adenosyl-L-methionine dependent methyltransferases.

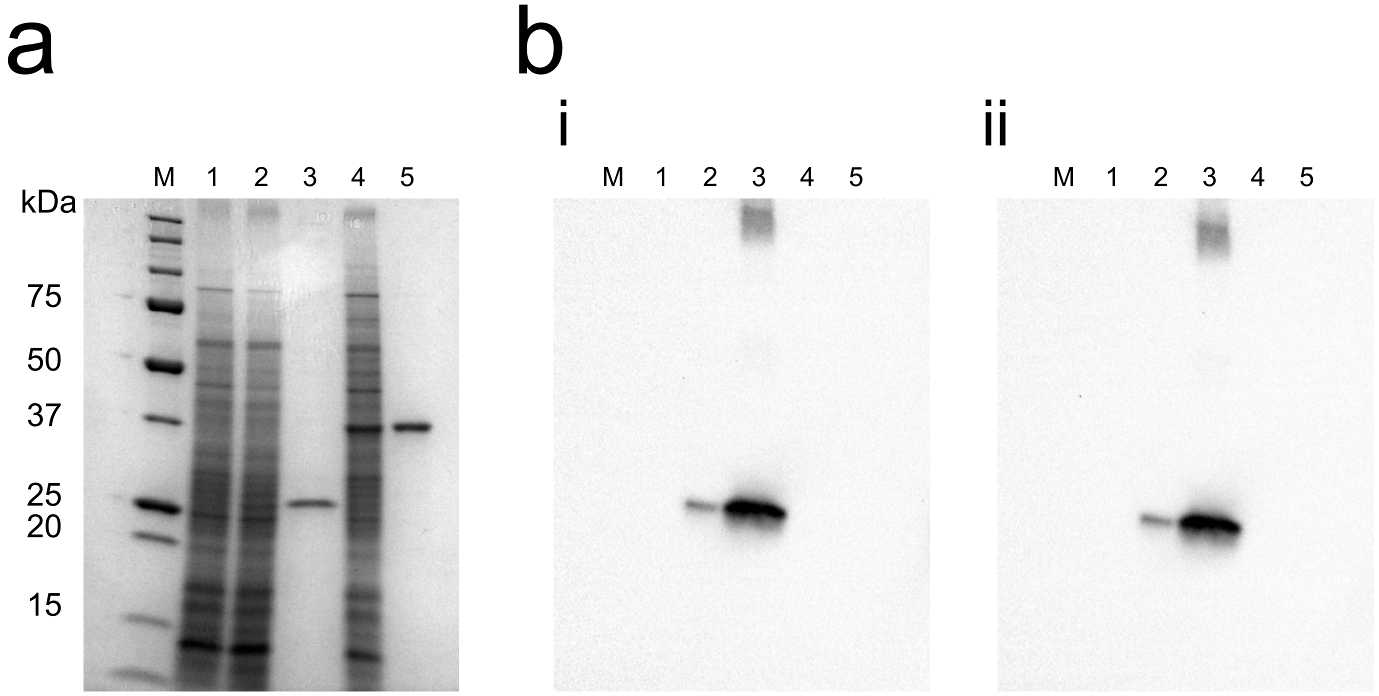

Supplementary Figure S1. SDS-PAGE and Western blotting analyses of His-tagged recombinant MySEV SAM-dependent MTase and *Mythimna separata* JHAMT expressed using the baculovirus protein expression system. (a) Full-length gel image of SDS-PAGE analysis. (b) Full-length blot images of Western blotting analysis. (i), exposure time: 48.6 seconds. (ii), exposure time: 70.0 seconds. An anti-MySEV SAM-dependent MTase peptide antiserum was used for Western blotting analysis. Lane 1, Lysate of cells infected with viruses that have no target gene. Lane 2, Lysate of cells infected with viruses expressing the MySEV SAM-dependent MTase gene. Lane 3, Purified His-tagged MySEV SAM-dependent MTase protein. Lane 4, Lysate of cells infected with viruses expressing the M. separata JHAMT gene. Lane 5, Purified His-tagged *M. separata* JHAMT. Lane M, protein standard marker.

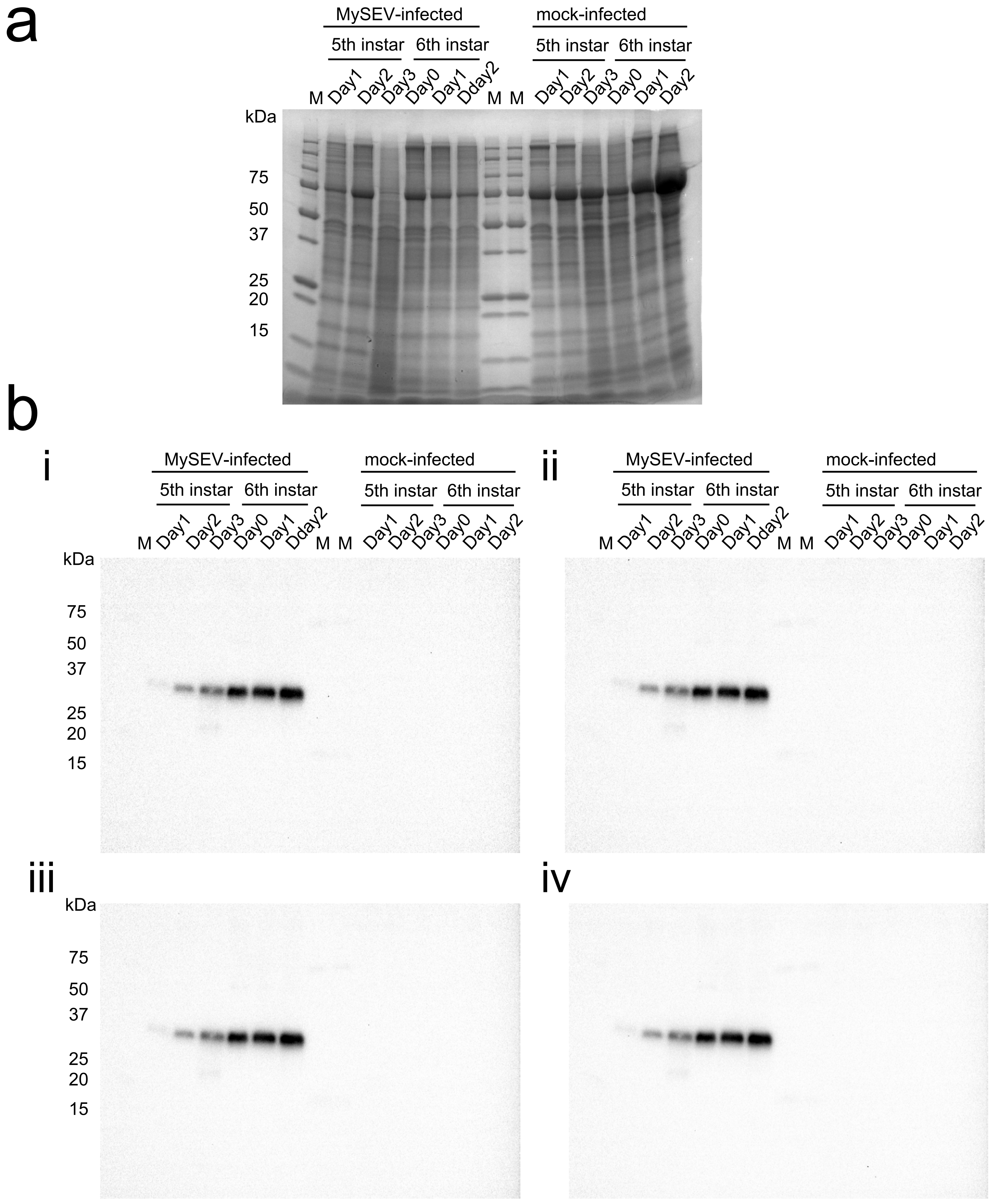

Supplementary Figure S2. SDS-PAGE and Western blotting analyses of MySEV SAM-dependent MTase in the hemolymph of larval *M. separata*. (a) Full-length gel image of SDS-PAGE analysis. (b) Full-length blot images of Western blotting analysis. (i), exposure time: 24.8 seconds. (ii), exposure time: 39.1 seconds. (iii), exposure time: 53.3 seconds. (iv), exposure time: 65.8 seconds. Larvae were MySEV- or mock-infected at 4th instar, and hemolymph was collected from 5th instar to early 6th instar. Anti-MySEV SAM-dependent MTase peptide antiserum was used for Western blotting analysis. Lane M, protein standard marker.

a

Information on primers used in determining an internal control gene for real-time PCR

Gene	GenBank accession #	Sequence	Product length (bp)	Efficiency (%)	r^2
β-actin (*ACT*)	GQ856238	GCCGCGACCTCACAGACTAC<br>CGAGAGCGACGTAGCAGAGC	120	106.4	0.994
Elongation factor 1 alpha (*EF1*)	KR869784	GAAACTGGCTTCGACAGAGG<br>GACTGTTGGGTCGTCCATCT	111	94.2	0.985
Ribosomal protein S11e (*Rp11e*)	GQ222274	GGCAATTGAAGGCACATACA<br>GTCACGTCTGATGACGATGG	127	97.3	0.997
Ribosomal protein L32 (*RpL32*)	AB669190	CTGGCGTAAACCCAGAGGTA<br>CATTGTGGACCAGCACTTTG	143	90.3	0.993
Ribosomal protein S7 (*RpS7*)	JN582331	ATCGAACTGCACAACAAGAAGTC<br>CGGTCTCCAACGAACACAA	140	96.4	0.997
Ribosomal protein S5 (*RpS5*)	LC310989	CGTTGAGCGTCTGACCAAC<br>CGTGCTTCACAATACGGACA	83	96.6	0.993
Ribosomal protein S2 (*RpS2*)	LC310988	AAGGATGATAGCACCACGGA<br>CGGAGACAACAACGGACA	82	95.9	0.992

b

Relative expression stability of selected reference genes in larval heads as determined by NormFinder

Control insects across 5th to 6th instar			EPV-infected insects across 5th to 6th instar			Control vs EPV-infected insects		
Rank	Gene	Stability value	Rank	Gene	Stability value	Rank	Gene name	Stability value
1	***RpS5***	0.179	1	***RpL32***	0.145	1	***RpS2***	0.064
2	***RpL32***	0.190	2	***RpS5***	0.155	2	***RpL32***	0.067
3	***RpS2***	0.205	3	***Rp11e***	0.168	3	***Rp11e***	0.070
4	***RpS7***	0.226	4	***EF1***	0.198	4	***RpS7***	0.082
5	***EF1***	0.232	5	***RpS2***	0.190	5	***RpS5***	0.091
6	***Rp11e***	0.240	6	***RpS7***	0.201	6	***EF1***	0.097
7	***ACT***	0.595	7	***ACT***	0.292	7	***ACT***	0.180

c

BestKeeper analysis of selected referemce genes in larval heads

Control insects					EPV-infected insects					Control and EPV-infected insects				
Rank	Gene	SD	*r*	*p*	Rank	Gene	SD	*r*	*p*	Rank	Gene	SD	*r*	*p*
1	***Rp11e***	0.544652127	0.957	0.001	1	***Rp11e***	0.443103758	0.957	0.001	1	***Rp11e***	0.498051771	0.954	0.001
2	***RpS7***	0.557834593	0.946	0.001	2	***RpS5***	0.48408483	0.95	0.001	2	***RpS7***	0.533379325	0.922	0.001
3	***RpL32***	0.589765084	0.968	0.001	3	***RpS7***	0.499611888	0.899	0.001	3	***RpS5***	0.550132963	0.956	0.001
4	***RpS5***	0.598679847	0.975	0.001	4	***RpL32***	0.512914289	0.963	0.001	4	***RpL32***	0.554960692	0.963	0.001
5	***EF***	0.827102255	0.959	0.001	5	***RpS2***	0.764202085	0.968	0.001	5	***RpS2***	0.796454103	0.972	0.001
6	***RpS2***	0.831728982	0.977	0.001	6	***EF1***	0.780373287	0.958	0.001	6	***EF***	0.808008872	0.955	0.001
7	***ACT***	0.945589021	0.954	0.001	7	***ACT***	0.815366273	0.911	0.001	7	***ACT***	0.88716421	0.918	0.001

Supplementary Figure S3. Validation of internal control genes for RT-qPCR of *M. separata JHAMT* expression in head tissues. (a) Information about primers used in the experiment. Efficiency (%) is an estimated measure of the real-time PCR efficiency utilizing standard curve methods. NormFinder analysis (b) and BestKeeper analysis (c) for relative expression stability of the selected reference genes. Lower stability values in NormFinder analysis and lower standard deviations in BestKeeper analysis mean higher expression stability. Genes with stability values of < 0.5 can be used for endogenous normalization. Standard deviations of > 1.0 indicate that the genes are unstably expressed. NormFinder showed that all genes were suitable for endogenous normalization except for the β-actin gene in mock-infected insects. *RpL32* was ranked in the top two in all the expression stability analyses of either mock-infected or EPV-infected insects during 5th to early 6th instar, and mock-infected insects vs. EPV-infected insects. BestKeeper showed that all genes were stable. *Rp11e* was always ranked as top 1 but *Rp11e*, *RpS7*, *RpS5*, and *RpL32* had similar standard deviation values.

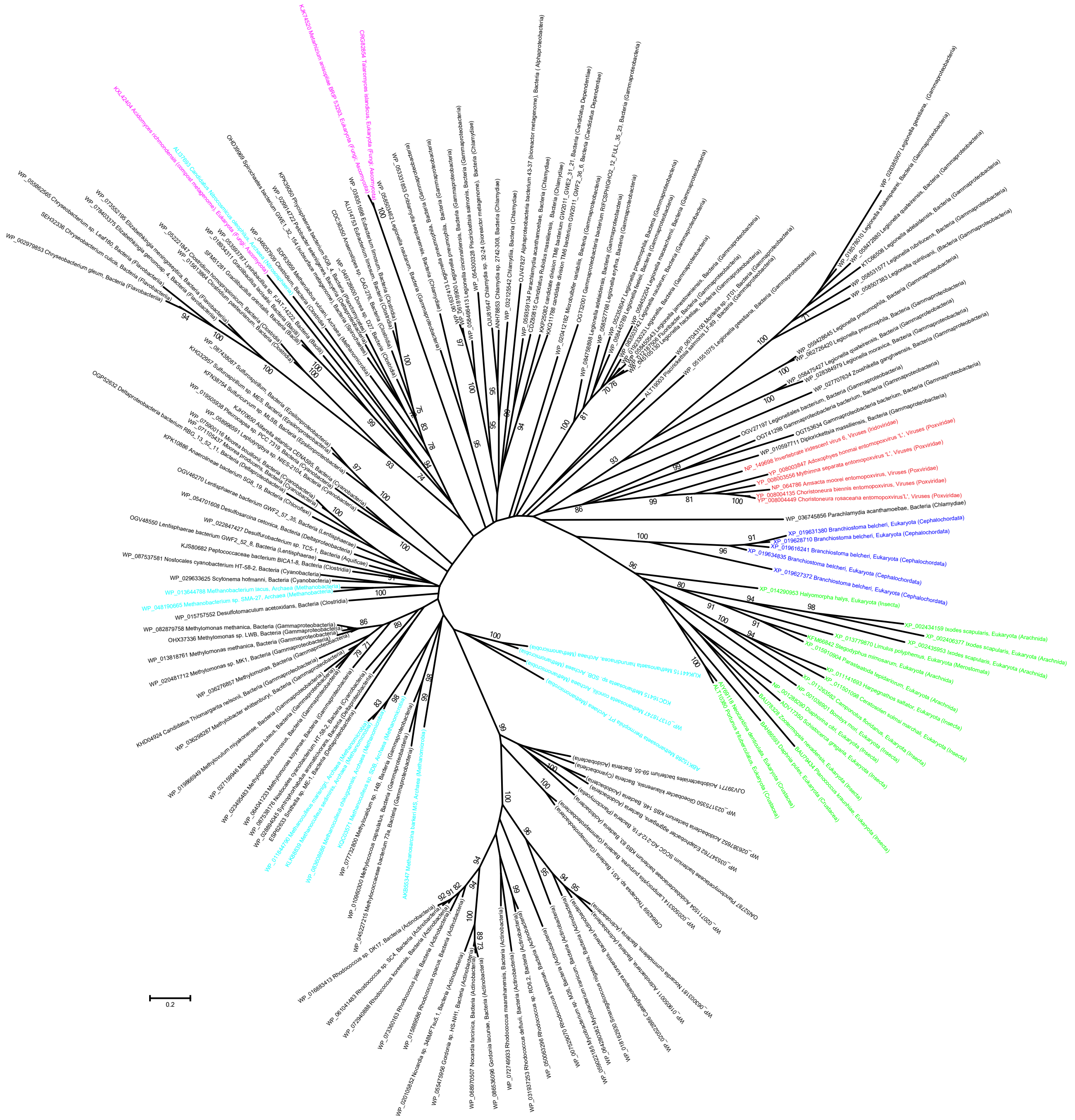

Supplementary Figure S4. Phylogenetic tree of SAM-dependent methyltransferases obtained by a maximum likelihood analysis of a multiple alignment of homologous proteins with MySEV JHAMT. Numbers at the nodes indicate bootstrap values of maximum likelihood analysis generated after 100 replicates. Branch termini are labeled with sources: red, viruses; green, arthropods; blue, amphioxi; pink, fungi; black, bacteria; cyan, archaea.

Supplementary Method

Selection of an internal control gene for RT-qPCR of *M. separata JHAMT* expression in head tissues

Quantitative RT-PCRs were performed for the same head tissue samples used in quantitative analysis of host *JHAMT*, targeting selected candidate genes (Supplementary Fig. S3). Quantification cycle values (i.e., cycle number estimated from the intersection of the threshold and a given amplification curve) were acquired in duplicate per sample. Three no-template controls and a duplicate 5-fold dilution series of standard were included in each run. Data were analyzed using NormFinder[1] and BestKeeper[2]. Linear scale values corresponding to the quantification cycle values were estimated from the standard curves for NormFinder analyses. Relative expression stability for each gene was estimated for biological samples across either mock-infected or EPV-infected insects during sampling times, and across mock-infected and EPV-infected insects in the analyses by NormFinder. In BestKeeper analysis, standard deviation and Pearson’s coefficient of correlation were calculated for expression of each gene across either mock-infected insects or EPV-infected insects during sampling times, and across mock-infected and EPV-infected insects.

References

1 Andersen, C. L., Jensen, J. L. & Ørntoft, T. F. Normalization of real-time quantitative reverse transcription-PCR data: a model-based variance estimation approach to identify genes suited for normalization, applied to bladder and colon cancer data sets. *Cancer Res.* **64**, 5245-5250, doi:10.1158/0008-5472.can-04-0496 (2004).

2 Pfaffl, M. W., Tichopad, A., Prgomet, C. & Neuvians, T. P. Determination of stable housekeeping genes, differentially regulated target genes and sample integrity: BestKeeper--Excel-based tool using pair-wise correlations. *Biotechnol. Lett.* **26**, 509-515, doi:10.1023/B:BILE.0000019559.84305.47 (2004).
